# Gamabufotalin induces a negative feedback loop connecting ATP1A3 expression and the AQP4 pathway to promote temozolomide sensitivity in glioblastoma cells by targeting the amino acid Thr794

**DOI:** 10.1111/cpr.12732

**Published:** 2019-11-20

**Authors:** Yu‐Long Lan, Cheng Chen, Xun Wang, Jia‐Cheng Lou, Jin‐Shan Xing, Shuang Zou, Ji‐Liang Hu, Wen Lyu, Bo Zhang

**Affiliations:** ^1^ Department of Neurosurgery Shenzhen People’s Hospital Second Clinical Medical College of Jinan University First Affiliated Hospital of Southern University of Science and Technology Shenzhen China; ^2^ Department of Neurosurgery The Second Affiliated Hospital of Dalian Medical University Dalian China; ^3^ Department of Physiology Dalian Medical University Dalian China

**Keywords:** AQP4, ATP1A3, gamabufotalin, glioblastoma multiforme, temozolomide

## Abstract

**Objectives:**

Temozolomide (TMZ) is one of the most commonly used clinical drugs for glioblastoma (GBM) treatment, but its drug sensitivity needs to be improved. Gamabufotalin (CS‐6), the primary component of the traditional Chinese medicine “ChanSu,” was shown to have strong anti‐cancer activity. However, more efforts should be directed towards reducing its toxicity or effective treatment doses.

**Methods:**

Target fishing experiment, Western blotting, PCR, confocal immunofluorescence and molecular cloning techniques were performed to search for possible downstream signalling pathways. In addition, GBM xenografts were used to further determine the potential molecular mechanisms of the synergistic effects of CS‐6 and TMZ in vivo.

**Results:**

Mechanistic research revealed a negative feedback loop between ATP1A3 and AQP4 through which CS‐6 inhibited GBM growth and mediated the synergistic treatment effect of CS‐6 and TMZ. In addition, by mutating potential amino acid residues of ATP1A3, which were predicted by modelling and docking to interact with CS‐6, we demonstrated that abrogating hydrogen bonding of the amino acid Thr794 interferes with the activation of ATP1A3 by CS‐6 and that the Thr794Ala mutation directly affects the synergistic treatment efficacy of CS‐6 and TMZ.

**Conclusions:**

As the main potential target of CS‐6, ATP1A3 activation critically depends on the hydrogen bonding of Thr794 with CS‐6. The combination of CS‐6 and TMZ could significantly reduce the therapeutic doses and promote the anti‐cancer efficacy of CS‐6/TMZ monotherapy.

## 1 INTRODUCTION

Glioma is the most common primary central nervous system (CNS) tumour and comprises approximately 60%‐70% of all primary brain tumours. Among them, glioblastoma (GBM) is the most malignant type.^1^ GBM has become an intractable challenge in neurosurgery, and currently, the main treatment method is surgery combined with radiotherapy and/or chemotherapy. However, due to the rapid growth and high invasiveness of GBM and the prevalence of drug resistance, new therapeutic targets and improvements in the prognosis of GBM patients via enhanced treatment strategies are urgently needed.

The traditional Chinese medicine ChanSu has been widely used for clinical cancer treatment in some countries.^2^ Currently, more than 100 bufadienolides, including gamabufotalin (CS‐6), bufalin, cinobufagin (CB), resibufogenin (RB) and telecinobufagin (CS‐7), have been separated and identified as the major active components with anti‐cancer activities in ChanSu.^3^ CS‐6, a major bufadienolide of ChanSu, has been used for cancer therapy due to its good metabolic stability and few adverse effects.^4^, ^5^, ^6^, ^7^ However, CS‐6 also has an equivalent killing effect on normal cells, which is a major problem that has not yet been solved. Therefore, if the drug could be altered to selectively kill cancer cells or show increased efficacy at low concentrations, research on the clinical applications of CS‐6 would be substantially accelerated. In addition, temozolomide (TMZ) is one of the most commonly used clinical drugs in glioma treatment, but recurrent drug resistance limits the clinical application of this drug. Thus, identification of compatible drugs that could promote the anti‐cancer effect of TMZ will substantially improve the treatment efficacy of patients with GBM.

Sodium pumps (Na^+^/K^+^‐ATPase) are widespread in eukaryotic cell membranes, and four different α isoforms (ATP1A1‐1A4) and three distinct β isoforms (ATP1B1‐1B3) have been identified in humans.^8^ In recent years, high expression of sodium pumps on the surface of mammal cell membranes was shown to be closely related to the occurrence, development and malignancy of cancer.^8^ Recent research has indicated that the expression of sodium pump α1 subunit protein (ATP1A1) in GBM cell lines and tissue samples was higher than that in normal tissues.^9^ Thus, ATP1A1 on the surface of GBM cell membranes might be a potential therapeutic target of anti‐cancer drugs,^10^ but more research is needed. Notably, ATP1A3 was reported to exert important effects in various cancers, including GBM,^11^ hepatoma and medulloblastoma.^12^, ^13^ Interestingly, Li et al^12^ indicated that ATP1A3 expression status may help predict the sensitivity of hepatocellular carcinoma (HCC) cells to bufalin (another kind of bufadienolide with pharmacological activity similar to CS‐6) treatment. In addition, these findings have various implications for human CNS diseases caused by ATP1A3 mutations,^14^ and ATP1A3 mutations have been identified in patients with multiple diseases, including alternating hemiplegia of childhood (AHC); rapid onset dystonia parkinsonism (RDP); cerebellar ataxia, areflexia, pes cavus, optic atrophy and sensorineural hearing loss (CAPOS) syndrome; relapsing encephalopathy with cerebellar ataxia (RECA); and fever‐induced paroxysmal weakness and encephalopathy (FECA).^15^, ^16^, ^17^ However, research on the roles of ATP1A3 in GBM is lacking and could contribute to the diagnosis, treatment and prognosis of GBM patients. Although ATP1A3 is a potential target of CS‐6, no crystal structure has been published. Thus, little is known about the mode in which CS‐6 binds to ATP1A3 and how this interaction ultimately results in activation of the channel protein. To identify amino acids interacting with CS‐6, we built a homology model of ATP1A3 and computationally docked CS‐6. Based on these studies, we chose amino acids in close proximity to and therefore potentially interacting with CS‐6, mutated the selected amino acids and evaluated the ability of these mutants to respond to CS‐6, as well as its combination with TMZ.

Thirteen aquaporins (AQP0‐AQP12) have been identified in mammals. Among them, aquaporin‐4 (AQP4), which is specifically expressed in glial cells, is the main aquaporin in the CNS. Currently, an increasing number of studies have indicated the important roles of AQP4 in the malignant growth of gliomas.^18^ The expression of AQP4 was shown to be increased in glioma, while AQP4 inhibition could significantly hinder glioma growth.^19^, ^20^ However, the mechanisms of AQP4 function in glioma still require more systematic and comprehensive research. Intriguingly, a recent study indicated that TMZ had therapeutic potential for suppressing malignant glioma by inhibiting AQP4 expression through activation of the p38 signal transduction pathway,^21^ indicating that screening for specific small‐molecule inhibitors of the AQP4‐MAPK pathway may contribute to the design of novel mechanism‐based therapies for GBM. In addition, the blood‐brain barrier (BBB) is known to play an important role in the resistance to chemotherapeutic drugs in glioma, and AQP4 plays an irreplaceable role in maintaining BBB integrity^22^; thus, AQP4 may be an important target for anti‐drug resistance strategies in glioma. In this study, we further elucidated the therapeutic effect of AQP4 in malignant glioma and its molecular mechanism in anti‐glioma treatments.

Currently, the correlation between ATP1A3 and AQP4 in human cancers and their mechanistic interactions have not been addressed. In the current study, we focused on the interaction between ATP1A3 and AQP4 in GBM cells, seeking to shed light on the effect of the feedback loop between ATP1A3 and AQP4 in controlling the growth of GBM and to help clarify their roles in promoting TMZ sensitivity. Furthermore, we used point mutations to identify the potential critical residues for ATP1A3 activation and the synergistic treatment effect of CS‐6 and TMZ. This approach will help elucidate the structural basis of ATP1A3 function during the addition of CS‐6 to TMZ.

## 2 MATERIALS AND METHODS

### 2.1 Antibodies and other materials

The primary antibodies for ATP1A3, ATP1A4, AQP4, O(6)‐methylguanine‐DNA methyltransferase (MGMT), GAPDH and β‐actin and all the secondary antibodies were obtained from Saituo Biotech Company (Dalian, China). Primary antibodies against Na^+^‐K^+^‐ATPase α1 (ATP1A1), p38, p‐p38 and cleaved PARP were obtained from ProteinTech Company. Trypsin, Dulbecco's modified Eagle's medium (DMEM) and foetal bovine serum (FBS) were obtained from HyClone Laboratories (HyClone Laboratories, Inc). CS‐6 (purity > 98%) was purchased from Yuanye Biotech Company, added to DMSO and kept at −20°C as a stock solution. CS‐6 was diluted in culture medium to obtain the desired concentration and was stable in DMSO. Phosphate‐buffered saline (PBS), protease inhibitor cocktail and 5‐diphenyltetrazolium bromide (MTT) were purchased from Sigma Chemical Company. All other chemicals were purchased from Sigma Chemical Company unless otherwise specified.

### 2.2 Cell culture

All cell lines were authenticated using short tandem repeat (STR) analysis by Shanghai GeneChem Co., Ltd., and the cell lines were confirmed to be free of mycoplasma by the Mycoplasma Detection Kit‐Quick Test (Biotool Biotechnology). U87, U251, LN229, LN18, A172 and T98 cells were cultured in DMEM supplemented with 15% FBS, while U118 cells were cultured in DMEM supplemented with 10% FBS, and all cell lines were grown at 37°C in a humidified atmosphere with 5% CO_2_.

### 2.3 Cell viability assay

Cell viability was determined by MTT assays (Roche Diagnosis, Indianapolis, IN, USA). MTT powder was dissolved, and after ultrasonic dissolution for 1 hour, the solution was adjusted to a final concentration of 5 mg/mL. Cells in a 96‐well plate were treated with drugs for 48 hours, followed by the addition of MTT solution with a final concentration of 0.5 mg/mL. Then, the plate was incubated at 37°C for 4 hours. After the culture medium was discarded, 150 μL DMSO was added into each well, and the plate was shaken for 10 minutes, followed by detection of the absorbance value of each well by an EnSpire® Multimode Plate Reader (Perkin Elmer) with a wavelength of 570 nm.

### 2.4 Target fishing experiment

This project strictly followed the relevant regulations of Plexera to conduct the experiment.^23^ The instrument, named PlexArray^®^ HT and Molecular Chip for Target fishing, was from Plexera LLC. After the surface plasmon resonance (SPR) experiment,^23^, ^24^ all target proteins were treated with trypsin after elution from the chip, and then, HPLC‐MS/MS identification experiments were carried out. The corresponding experimental parameters are given in Table S1. All target proteins were treated with trypsin after elution from the chip and then subjected to the HPLC‐MS/MS identification experiment.

Design of the fixed small Molecular Chip for Target fishing experiment: The chips were divided into four pieces, two of which were immobilized with CS‐6, and the other two were used as blank controls with immobilized methanol. CS‐6 was dissolved and then diluted to a concentration of 2.5 mmol/L with methanol. These two chips were equally saturated with 30 µL CS‐6 as a positive control. The other two chips were saturated with methanol (30 µL) as a negative control.

Design of flowing cell lysis: U87 and U118 cell lysates were diluted to 1 mg/mL and divided into two parts, one for the positive control and the other for the blank control.

Target fishing experiment process: SPR of target fishing was divided into two parts, and U87 cells and U118 cells were assessed in two groups of parallel control experiments. (a) Positive control: The U87/U118 cell lysate sample was flowed onto the chip saturated with CS‐6 installed on the SPR instrument, which could monitor and detect the target protein in the cell lysate captured by the small molecule on the chip in real time. Collection of target proteins from chips by glycine‐HCl: All the target proteins were treated with trypsin after elution from the chip, and then, an HPLC‐MS/MS identification experiment was carried out. (b) Blank control: The procedures were the same as above, and the sole difference was that the blank control chip was saturated with methanol.

### 2.5 Colony formation assay

Cells were spread in a 6‐well plate (1000/well), mixed and cultured in a CO_2_ incubator at constant temperature for 24 hours. Then, we replaced the previous culture media with new media containing the indicated concentrations of drug solution, and the cells were cultured for another 10‐12 hours. After PBS washes, new culture medium was added, and the cells were cultured for another 14 days. Then, after PBS washes, 1 mL stationary liquid was added into each well (glacial acetic acid:methanol:deionized water = 1:1:8) and shaken for 10 minutes. The colonies were counted after staining with 0.1% crystal violet.

### 2.6 Apoptosis assay

The apoptosis rates were determined by using an Annexin V‐FITC Apoptosis Detection Kit (Houston, TX, USA). Cells were placed in 6 cm culture vessels and grown to full confluence. After the indicated treatment for 12 hours, cells were washed three times and digested with trypsin without EDTA. After being suspended in 0.2% BSA, the cells were centrifuged (600 g/min, 5 minutes), and 500 µL binding buffer was added to the cell pellet in the dark, followed by the addition of 5 µL Annexin V‐FITC and 5 µL propidium iodide in the dark. After 5‐15 minutes at room temperature in the dark, the fraction of apoptotic cells was determined using a FACS analysis system (BD FACS Accuri C6).

### 2.7 Cell cycle analysis by flow cytometry

The cells were grown to full confluence in a 6‐well plate and starved for 4 hours. After the indicated treatments, the cells were washed and digested by trypsin and suspended in PBS. Next, 1200 µL of 70% ethanol was added for fixation for 2 hours. Then, the cells were centrifuged (1000 *g*, 5 minutes, 4°C), followed by suspension with 1 mL PBS. After addition of 80 µL RNase A and mixing, the samples were placed at 37°C for 30 minutes, and 320 µL PI was added and mixed, followed by incubation at 4°C in the dark for 30 minutes. Finally, flow cytometry analysis was performed by using a flow cytometer (BD FACS Accuri C6).

### 2.8 Western blotting

Protein concentrations of collected cell precipitations were determined by using a protein concentration determination kit (Beyotime Biotechnology). After denaturation, proteins (50 μg) were applied to 8% to 12% SDS‐PAGE gels and transferred onto a PVDF membrane (Millipore). After the PVDF membrane was incubated with the indicated antibodies, the protein bands were detected by enhanced chemiluminescence. The bands were normalized to those of β‐actin or GAPDH as an internal control.

### 2.9 Real‐time reverse transcription polymerase chain reaction (RT‐PCR)

With an RNA extraction kit (TaKaRa Bio), total RNA was extracted from cells using TRIzol reagent, followed by reverse transcription of cDNA by using the PrimeScript RT Reagent Kit (TaKaRa Bio). The primer pairs were as follows: ATP1A3, Forward: 5'‐TGGGAGGAGGAGAGGAACAG‐3' and Reverse: 5'‐GATGGAGAAGCCCCCGAAG‐3'; and β‐actin, Forward: 5'‐GGCACCCAGCACAATGAA‐3' and Reverse: 5'‐TAGAAGCATTTGCGGTGG‐3'. The amplification products were analysed using 1.5% agarose gel electrophoresis and stained with ethidium bromide for visualization using ultraviolet light.

### 2.10 ATP1A3 knockdown using short hairpin RNAs (shRNAs)

The lentiviral vectors were purchased from Synbio Tech (Suzhou). ATP1A3 shRNA (shRNA‐ATP1A3) or negative control shRNA (shRNA‐NC) was inserted into the PLKO.1 vector. For generation of the lentiviruses, Rev, Gag, VSVG and plasmid were introduced into 293T cells. Then, we harvested the supernatant at 48 hours post‐transfection and filtered it with a 0.45 μm membrane. For virus infection, the cancer cell medium was treated with virus supernatant at a 1:5 ratio, and after 24 hours, we used ~2 μg/mL puromycin to select the ATP1A3‐suppressed stable cell lines. The surviving cells were used for the subsequent experiments. Three pairs of shRNA sequences targeting the ATP1A3 gene were designed and synthesized: sh1 (F: 5'‐ CCGGCGACGAAATCCGCAAACTCATCTCGAGATGAGTTTGCGGATTTCGTCGTTTTTG‐3'; R: 5'‐ AATTCAAAAACGACGAAATCCGCAAACTCATCTCGAGTTTATGCTTGGAATCATTTGG‐3'); sh2 (F: 5'‐ CCGGGCCTTCTTTGTGAGCATCGTTCTCGAGAACGATGCTCACAAAGAAGGCTTTTTG‐3'; R: 5'‐ AATTCAAAAAGCCTTCTTTGTGAGCATCGTTCTCGAGAACGATGCTCACAAAGAAGGC‐3'); and sh3 (F: 5'‐ CCGGCAAACCTCTCTCCTCTCTCTTCTCGAGAAGAGAGAGGAGAGAGGTTTGTTTTTG‐3'; R: 5'‐ AATTCAAAAACAAACCTCTCTCCTCTCTCTTCTCGAGAAGAGAGAGGAGAGAGGTTTG‐3'). A non‐silencing shRNA (5'‐GCCTAACTGTGTCAGAAGGAA‐3') was used as the negative control.

### 2.11 AQP4 knockdown using shRNAs

The lentiviral vectors were purchased from Synbio Tech (Suzhou). Two pairs of shRNA sequences targeting the AQP4 gene were designed and synthesized: sh1 (F: 5'‐AGACUUGGCGAUGCUGAUCTT‐3'; R: 5'‐GAUCAGCAUCGCCAAGUCUTT‐3') and sh2 (F: 5'‐GAUCCUCUACCUGGUCACATT‐3'; R: 5'‐UGUGACCAGGUAGAGGAUCTT‐3'). A non‐silencing shRNA (5'‐GCCTAACTGTGTCAGAAGGAA‐3') was used as the negative control.

### 2.12 ATP1A3 overexpression via transfection by using lentiviral vectors

The lentiviral vectors were purchased from YouBio (Changsha, Hunan, China). The ATP1A3 cDNA sequence was cloned and ligated into the pCDH‐GFP‐PURO‐3xFlag vector. For generation of lentiviruses, 245, 246 and plasmid were introduced into 293T cells. Then, we harvested the supernatant at 48 hours post‐transfection and filtered it with a 0.45 μm membrane. For virus infection, the cell medium was treated with virus supernatant at a 1:5 ratio, and after 24 hours, we used ~s2 μg/mL puromycin (Sigma) to select the ATP1A3‐overexpressing stable cell lines. The primers for ATP1A3 were designed and synthesized: Forward, 5'‐ AGGTCGACTCTAGAGGATCCCGCCACCATGGGGGACAAGAAAGATGACAAGGAC‐3'; Reverse, 5'‐ TCCTTGTAGTCCATACCGTAGTAGGTTTCCTTCTCCACCCAACC‐3'. The ATP1A3‐overexpressing cells were named lenti‐ATP1A3; the corresponding control cells were named lenti‐vector.

### 2.13 AQP4 overexpression via transfection by using lentiviral vectors

The lentiviral vectors were purchased from YouBio (Changsha). The AQP4 cDNA sequence was cloned and ligated into the pCDH‐GFP‐PURO‐3xFlag vector. For generation of the lentiviruses, 245 and 246 plus transfer vectors were introduced into 293T cells. Then, we harvested the supernatant at 48 hours post‐transfection and filtered it with a 0.45 μm membrane. For virus infection, the cell medium was treated with virus supernatant at a 1:5 ratio, and after 24 hours, we used ~2 μg/mL puromycin (Sigma) to select the AQP4‐overexpressing stable cell lines. The primers for AQP4 were designed and synthesized: Forward, 5'‐GCTCTAGAATGAGTGACAGACCCACAGCAAG‐3'; Reverse, 5'‐ATTTGCGGCCGCTCATACTGAAGACAATACCTCTC‐3'. The AQP4‐overexpressing cells were named lenti‐AQP4; the corresponding control cells were named lenti‐vector.

### 2.14 Site‐directed mutations for ATP1A3 (Thr794Ala, Ile312Ala, Leu790Ala)

Site‐directed mutagenesis of ATP1A3 was achieved using a one‐step PCR method. The mutations Thr794Ala, Ile312Ala and Leu790Ala were introduced into pIRES2‐EGFP‐TRPM2 by the QuikChange Site‐Directed Mutagenesis Kit (Agilent Technologies) using appropriate primer pairs. The presence of the mutation in the resulting plasmids was confirmed by sequencing the targeted region. Primers for ATP1A3 mutagenesis were designed and synthesized:

Thr794Ala:

Forward, 5'‐CTGCCCCTGGGCGCCATCACCATCCTCTGCATCGATC‐3';

Reverse, 5'‐ATGGTGATGGCGCCCAGGGGCAGCGGGATGTTGGCC‐3'.

Ile312Ala:

Forward, 5'‐TTGAGGCTGTCGCCTTCCTCATCGGCATCATCGTGG‐3';

Reverse, 5'‐CGATGAGGAAGGCGACAGCCTCAAGCCAGGTGTATC‐3'.

Leu790Ala:

Forward, 5'‐CAACATCCCGGCGCCCCTGGGCACCATCACCATCC‐3';

Reverse, 5'‐GTGCCCAGGGGCGCCGGGATGTTGGCCATGATGAAC‐3'.

The recombinant plasmid containing the original gene encoding ATP1A3 was used as the template. Routine PCR was performed as follows: 1 cycle at 94°C for 5 minutes, 35 cycles at 94°C for 30 seconds, 58°C for 1 minutes and 72°C for 1 minutes, and 72°C for last 10 minutes, using Taq Plus DNA polymerase. The amplified PCR products were processed using the DpnI restriction enzyme, and the nucleotide sequences of these mutant variants were verified by DNA sequencing by GeneChem.

### 2.15 Confocal immunofluorescence

After treatment with paraformaldehyde for 30 minutes, the samples were treated with Triton X‐100 for 2‐5 minutes. Then, the samples were blocked with blocking solution (1% bovine serum albumin and 2% glycerol in PBS) for 30 minutes and incubated with the primary antibodies against ATP1A3 or AQP4 diluted in PBS containing 1% BSA overnight at 4°C. After PBS washes, secondary fluorescein isothiocyanate‐conjugated antibodies were added for 1 hour at room temperature. Then, the cells were stained with 4',6‐diamidino‐2‐phenylindole (DAPI) (Beyotime, China) to stain the cell nuclei. Finally, the samples were examined with a Leica DM 14000B confocal microscope.

### 2.16 Immunofluorescence histochemical staining

The brain tissues were post‐fixed in 4% paraformaldehyde for 2 days. Sequential 15%‐30% sucrose treatment was performed for 2 days, and the brain samples were cryosectioned (16 mm thickness). The brain sections were washed in PBS 3 times. After blocking with 5% BSA solution for 1 hour, the brain sections were incubated with primary antibody at 4°C overnight. Alexa Fluor 488 or 594 secondary antibody (Invitrogen) was added for 1 hour. DAPI was used as the nuclei counterstaining dye. Then, we performed conventional or confocal imaging by using a Leica DM 4000B microscope or Leica TCS SP5 microscope.

### 2.17 Co‐immunoprecipitation (Co‐IP)

Cell extract proteins (400 μg) were precleared by adding 1 μg normal rabbit IgG and 30 μL protein A/G‐agarose beads (Santa Cruz Biotechnology). Together, they were incubated at 4°C for 1 hour with gentle agitation and centrifuge. The supernatants were collected and incubated with a specific mouse anti‐ATP1A3 antibody at 4°C for 5‐6 hour. The immune complexes were pulled down by protein A/G‐agarose beads and washed with ice‐cold PBS buffer containing proteinase inhibitor 3 times. After the final wash, the immune complexes were released by boiling in 2× loading buffer for 5 minutes, followed by Western blotting analysis with mouse anti‐ATP1A3 and rabbit anti‐AQP4 antibodies.

### 2.18 Biotinylation of cell surface proteins

Cells (3 × 10^5^) were seeded into T25 cell culture flasks and kept at 37°C and 5% CO_2_ for 3 days. Then, the cells were transfected with 7.5 mg of pIRES2‐EGFP, pIRES2‐EGFP‐ATP1A3 or one of the vectors modified by QuikChange mutagenesis using Lipofectamine LTX with PLUS reagent (Invitrogen) according to the manufacturer's protocol. Transfection was confirmed 24 hours after transfection by EGFP expression. At 48 hours post‐transfection, cells were washed with PBS and incubated with 1 mg/mL EZ‐Link Sulfo‐NHS‐LC‐Biotin (Pierce/Thermo Scientific) at room temperature for 30 minutes. After detachment and collection of the labelled cells, membrane proteins were isolated using the ProteoExtract Native Membrane Protein Extraction Kit (EMD Millipore) according to the manufacturer's protocol. For pulldown of biotinylated proteins, 50 mL of NeutrAvidin Agarose Beads (50% aqueous slurry; Pierce/Thermo Scientific) and 400 mg of the membrane proteins were incubated overnight using overhead rotation. Total membrane protein samples (10 μg each) and biotinylated protein samples after pulldown (from 400 mg of membrane protein) were separated by 7.5% SDS‐PAGE and transferred onto PVDF membranes. The membrane was probed for ATP1A3 or Na^+^/K^+^‐ATPase by incubation with anti‐ATP1A3 or Na^+^/K^+^‐ATPase antibody (Abcam, Dalian, China) and HRP‐conjugated goat anti‐rabbit IgG secondary antibody. Membranes were incubated for 5 minutes with a 1:10 mixture of SuperSignal West Dura chemiluminescent substrate (Pierce/Thermo Scientific) and SuperSignal West Pico chemiluminescent substrate (Pierce/Thermo Scientific), and chemiluminescence was detected using a LAS‐3000 Intelligent Dark Box (Fujifilm).

### 2.19 Molecular modelling

The experimental process had two parts: (a) homology modelling was conducted by using Molecular Operating Environment (MOE; Chemical Computing Group Inc) v2018.01. The template structure was identified through BLAST and was downloaded from the RCSB Protein Data Bank (PDB code: 4HYT). First, the target sequence was aligned to the template sequence, and ten independent intermediate models were built. These different homology models were the result of the permutational selection of different loop candidates and side‐chain rotamers. Then, the intermediate model that scored best according to the generalized Born/volume integral (GB/VI) solvation energy was chosen as the final model and was subjected to further energy minimization using the AMBER10/EHT force field. The protonation states of the protein and the orientations of the hydrogens were optimized by LigX at a pH of 7 and a temperature of 300 K. (b) Protein‐ligand docking: The 2D structure of the small molecule CS‐6 was drawn in ChemBioDraw 2014 and was converted to 3D conformations through energy minimization. The 3D structure of the protein ATP1A3 was downloaded from the RCSB Protein Data Bank (PDB code 4HYT). The docking process was performed under the force field of AMBER10/EHT along with an internal dielectric constant of 1 and an external dielectric constant of 80, as well as an implicit solvation model of the reaction field (R‐field). The Triangle Matcher algorithm was used for the initial placement of 1000 returned conformations, and the top 100 conformations ranked by London dG scoring function were further refined through energy minimization followed by rescoring using GB/VI, a more accurate implicit solvent model scoring function. The induced‐fit protocol was applied for energy minimization, in which the ligand was fully flexible during the conformation sampling process, and the side chains of the receptor were also allowed to move. Finally, 16 top‐ranked poses were retained, and the most representative pose was retrieved by visual inspection for further analysis.

### 2.20 Molecular dockings before and after the Thr794Ala mutation

The experimental process had two parts. (a) Construction of the ATP1A3 Thr794Ala, Ile312Ala and Leu790Ala mutants: We first opened the original PDB file using PyMOL (PyMOL version 2.1.0), then opened the Wizard‐>Mutagenesis panel, reselected the mutation locus and switched the amino acid to the target amino acid. Finally, we opened a File‐>Export Molecule panel to export the mutated protein. (b) Protein‐ligand docking: The Schrödinger Maestro package was employed to perform dock analysis before and after the point mutations. The 2D ligand structure of molecule CS‐6 was downloaded from PubChem (https://pubchem.ncbi.nlm.nih.gov) and prepared using LigPrep. The protein structure was modelled with I‐TASSER^25^ and constructed following the Protein Prepare Wizard workflow in the Maestro package. All chains of the structure were used to prepare the receptor, and all water molecules above 5 angstroms around the protein were removed. Then, potential allosteric sites of ATP1A3 were predicted by AllositePro,^26^ which will be described in the following text. The prepared ligand was then flexibly docked onto the predicted binding site of the receptor using Glide (XP mode) with default parameters. Finally, several docking poses were obtained for the molecule, and the ones with the best Glide scores were chosen before and after the point mutation.

### 2.21 Allosteric site prediction

AllositePro is a method that predicts allosteric sites in proteins by combining pocket features with perturbation analysis. The feature‐based model is trained on a high‐quality benchmarking data set, ASBench,^27^ by using a logistic regression method. Then, normal‐mode analysis (NMA), an efficient way to study probable cooperative motions of biomolecules, was used to evaluate the protein dynamic changes triggered by allosteric ligands. The score of the perturbation method perturbed NMA (PNMA) is defined by the p value in the Wilcoxon‐Mann‐Whitney test. In the final model, an allosteric site was identified by combining the two methods.

### 2.22 Human tissue microarray and immunohistochemistry (IHC)

Human tissue microarrays were purchased from Outdo Biotech Company. A total of 77 glioma tissue samples and 3 normal samples were included. Slides were deparaffinized, rehydrated and then immersed in target retrieval solution (pH 6) and boiled at medium heat three times for 10 minutes each in a microwave. After the slides were blocked with 3% BSA, the sections were incubated with primary antibody against AQP4 (Santa Cruz Biotechnology, 1:10 dilution) followed by HRP‐labelled anti‐rabbit IgG secondary antibody. The specimens were counterstained with haematoxylin. The negative control was obtained by replacing the primary antibody with a regular rabbit IgG. Target‐positive cells were counted in 3‐4 different fields and imaged using an Olympus microscope.

### 2.23 Animal studies

Ten microlitres of cell suspension containing a total of 1 × 10^6^ U87 cells was stereotactically implanted under the skin. After 10 days, the animals were stratified into the following groups: (a) drug vehicle, (b) 1 mg/kg CS‐6, (c) 20 mg/kg TMZ and (d) 1 mg/kg CS‐6 + 20 mg/kg TMZ. Treatment was intraperitoneally administered three times per week. Animals were sacrificed at the onset of symptoms using CO_2_. Tumours were removed and either formalin‐fixed for histology or snap‐frozen in liquid N_2_ for further studies. For determination of ATP1A3 and AQP4 expression, IHC was performed. The sections (4 μm) were stained with the ATP1A3 antibody (1:4000) and the AQP4 (1:10) antibody and examined under a light microscope. The images were examined under a Leica DM 4000B microscope.

Besides, for obtaining orthotopic xenografts to comparatively analyse the expressions of ATP1A3 or AQP4 in tumour and normal tissue, mice at 7 months of age were injected with U87 cells into the right hippocampi. On day 30 after tumour cell inoculation, mice were anaesthetized and perfused with ice‐cold PBS. Samples were harvested for biochemical or histological analysis.

All procedures were performed in accordance with the National Institutes of Health Guide for the Care and Use of Laboratory Animals (National Institutes of Health). All animals were purchased from the Experimental Animal Center, Dalian Medical University [Certificate of Conformity: No. SYXK (Liao) 2013‐0006]. The animals were acclimated to laboratory conditions (23°C, 12/12 h light/dark cycle, 50% humidity, with ad libitum access to food and water) for 2 weeks prior to the experiments, and no animals died before the experiments. In the animal study, all efforts were made to minimize the suffering of the mice.

### 2.24 Statistical analysis

Statistical analyses were performed using Student's *t* test or the nonparametric Mann‐Whitney U test (for the results of the Western blotting analyses). GraphPad Prism 6.0 software was used for statistical analyses. All data are presented as the mean ± standard error. *P* values less than .05 were considered significant: *P < .05, **P < .01, ***P < .001 and ****P < .0001.

## 3 RESULTS

### 3.1 CS‐6 improves TMZ sensitivity in glioma cells regardless of MGMT expression and ATP1A1 expression

**Figure 1:**
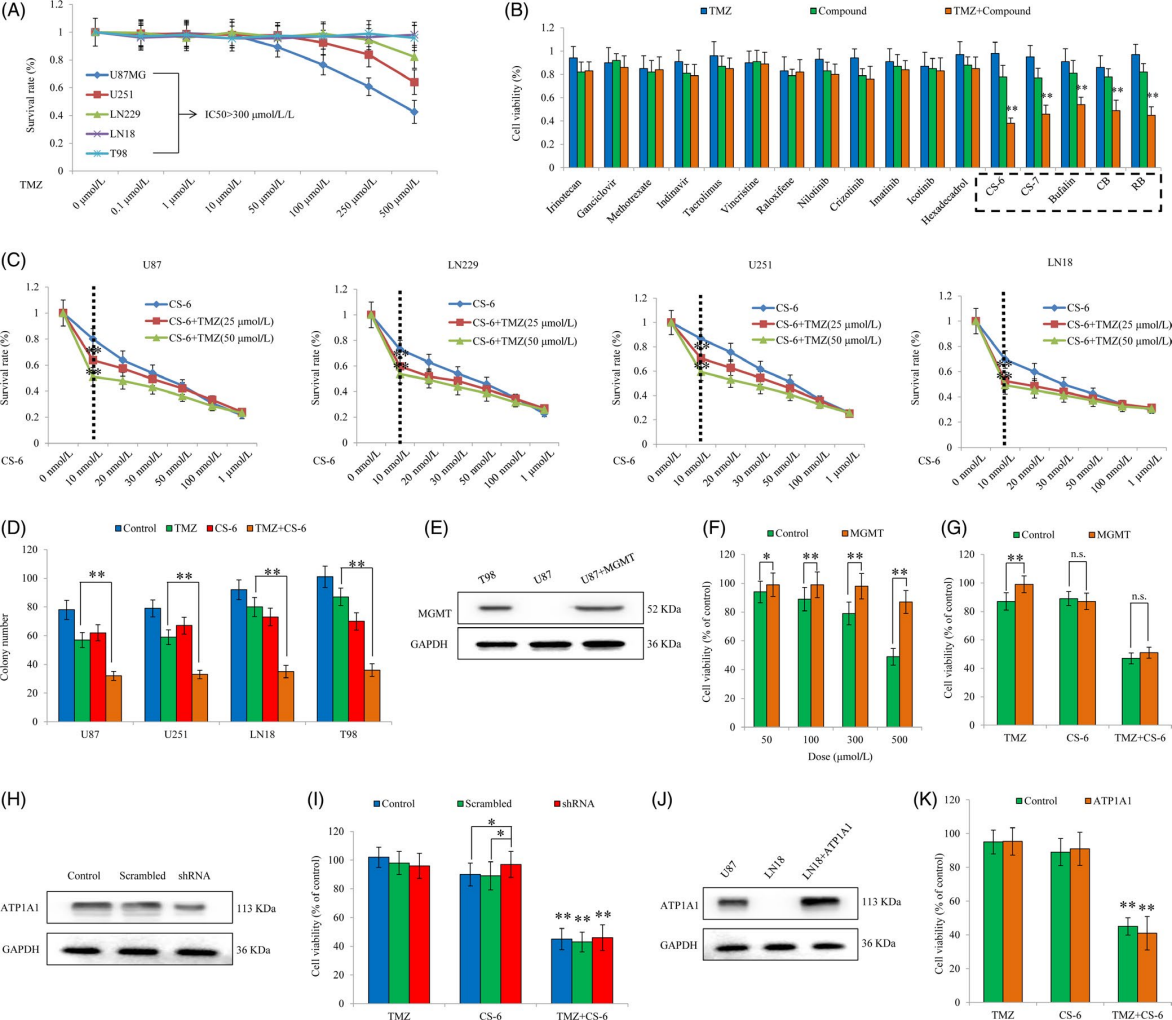
Identification of the chemosensitizing effect of CS‐6 in vitro. A, Effects of TMZ on the proliferation of various GBM cell lines. Cancer cells (U87, U251, LN229, LN18 and T98) were cultured with the indicated concentrations of TMZ for the indicated time, and then, the cell viability was determined. B, Identification of drugs potentiating the cytotoxic effect of TMZ. Twelve commonly used anti‐cancer drugs and 5 common active components of ChanSu were used in cytotoxicity assays in vitro. Each compound was tested at incremental doses as a monotherapy and in combination with 50 μmol/L TMZ. The figure shows cell viability in response to TMZ (left bar), the tested compound (middle bar) and the combination (right bar) after 96 h of drug treatment. C, After the addition of different concentrations of CS‐6 to 25 μ μmol/L /50 μ μmol/L TMZ for 48 h, a cell viability assay was performed to determine the synergistic effect of CS‐6 and TMZ, as well as the most appropriate CS‐6 concentration that could be used in the following experiments. D, Colony formation assays measured the cellular survival of TMZ‐sensitive (U87 and U251) and TMZ‐resistant (LN18 and T98) GBM cells in response to TMZ, CS‐6 and the combination of TMZ and CS‐6. Cells were treated with 50 μmol/L TMZ. All three cell lines were treated with 10 nmol/L CS‐6 either alone or in combination with TMZ for 96 h. E, Validation of MGMT expression in transfected U87 cells overexpressing MGMT. T98 cells were used as a positive control for MGMT expression. F, Cell viability assays indicating the response of U87 cells expressing MGMT and control cells to increasing concentrations of TMZ and (G) the response to 50 μmol/L TMZ, 10 nmol/L CS‐6 and the combination of 50 μmol/L TMZ and 10 nmol/L CS‐6 after 96 h of drug treatment. H, Western blotting indicating stable downregulation of ATP1A1 expression in U87 cells using an shRNA‐targeting strategy. I, Viability assays of U87 cells with established downregulation of ATP1A1 expression and control cells treated with 50 μmol/L TMZ, 10 nmol/L CS‐6 or the combination of 50 μmol/L TMZ and 10 nmol/L CS‐6 for 96 h. J, Confirmation of ATP1A1 expression in transfected LN18 cells overexpressing ATP1A1 protein. K, Viability assays of LN18 cells with or without ATP1A1 expression [as shown in (J)] after treatment with 50 μmol/L TMZ, 10 nmol/L CS‐6 or the combination of 50 μmol/L TMZ and 10 nmol/L CS‐6. Data are presented as the mean ± SEM of three independent experiments. *P < .05, **P < .01

We first tested the inhibitory effect of TMZ on the proliferation of our panel of GBM cells. We found that the IC_50_ values of TMZ for various cell lines generally exceeded 300 μmol/L, and no cytotoxic effect could be detected even at high concentrations (over 500 μmol/L) for LN18 and T98 (Figure 1A). We used 12 common clinical anti‐cancer drugs, as well as 5 commonly used compounds from ChanSu, in combination with TMZ to explore their synergistic anti‐cancer effects. The concentrations are shown for all clinical compounds that were used in combination with TMZ: 20 μmol/L irinotecan, 200 μmol/L ganciclovir, 100 μmol/L methotrexate, 200 μmol/L indinavir, 60 μmol/L tacrolimus, 0.6 μmol/L vincristine, 60 μmol/L raloxifene, 10 μmol/L nilotinib, 5 μmol/L crizotinib, 60 μmol/L imatinib, 200 μmol/L icotinib and 200 μmol/L hexadecanol. The results showed that 20 nmol/L CS‐6, 20 nmol/L bufalin, 30 nmol/L telecinobufagin (CS‐7), 50 nmol/L cinobufagin (CB) and 200 nmol/L resibufogenin (RB) significantly decreased cell viability when combined with TMZ compared to monotherapy or TMZ alone (**P < .01). The results indicated that some compounds had no therapeutic effect, whereas other compounds, including all 5 compounds from ChanSu, showed strong synergistic effects with concomitant TMZ therapy (Figure 1B). Among them, CS‐6 showed the best treatment effect when combined with TMZ; thus, we selected this drug for further research. We used different concentrations of CS‐6 in combination with TMZ at 20 μmol/L or 50 μmol/L, and cell viability assays were further used to examine the synergistic therapeutic effects. Interestingly, we found that CS‐6 at 10 nmol/L showed the most prominent chemosensitizing effect for TMZ therapy (Figure 1C).

To further confirm the effect of CS‐6 on the TMZ sensitivity of GBM cells, we expanded our research using various GBM cell lines with different genetic backgrounds. To further confirm the results of the cell proliferation assay, we performed a colony formation assay using U87, U251 (MGMT‐deficient), LN18 and T98 cell lines (the latter two cell lines expressing MGMT) to test the TMZ/CS‐6 sensitivity of the GBM cells. In both the MGMT‐deficient and MGMT‐proficient cell lines, CS‐6 cotreatment with TMZ notably led to a more pronounced decrease in tumour growth than monotherapy (Figure 1D). Subsequently, we further explored whether MGMT affected the synergistic treatment effect of TMZ and CS‐6. We found that transfected U87 cells overexpressing MGMT (Figure 1E) showed increased resistance to TMZ therapy (Figure 1F); however, MGMT did not impact the therapeutic effect of CS‐6 or the synergistic effect of CS‐6 and TMZ (Figure 1G). Importantly, MGMT expression did not determine the chemosensitizing effect of CS‐6.

To further identify the mechanisms underlying the chemosensitizing effect of CS‐6 as a cotreatment with TMZ, we next investigated the potential effect of ATP1A1. Our previous research suggested that bufadienolides could inhibit GBM via promoting ATP1A1 degradation.^28^ Thus, we examined whether ATP1A1 impacts the synergistic effect of TMZ and CS‐6. We knocked down ATP1A1 in U87 cells by using an shRNA strategy (Figure 1H); however, suppressing ATP1A1 in U87 cells did not affect the synergistic therapeutic effect of CS‐6 and TMZ (Figure 1I). We also overexpressed ATP1A1 in LN18 cells (Figure 1J), but the results showed that in LN18‐ATP1A1‐expressing cells, no detectable change in TMZ/CS‐6 sensitivity was found (Figure 1K). Taken together, our findings demonstrated that ATP1A1 expression does not modulate TMZ or CS‐6/TMZ sensitivity and that CS‐6 improves TMZ cytotoxicity regardless of MGMT or ATP1A1 expression.

### 3.2 CS‐6 suppresses GBM cell growth by targeting ATP1A3

**Figure 2:**
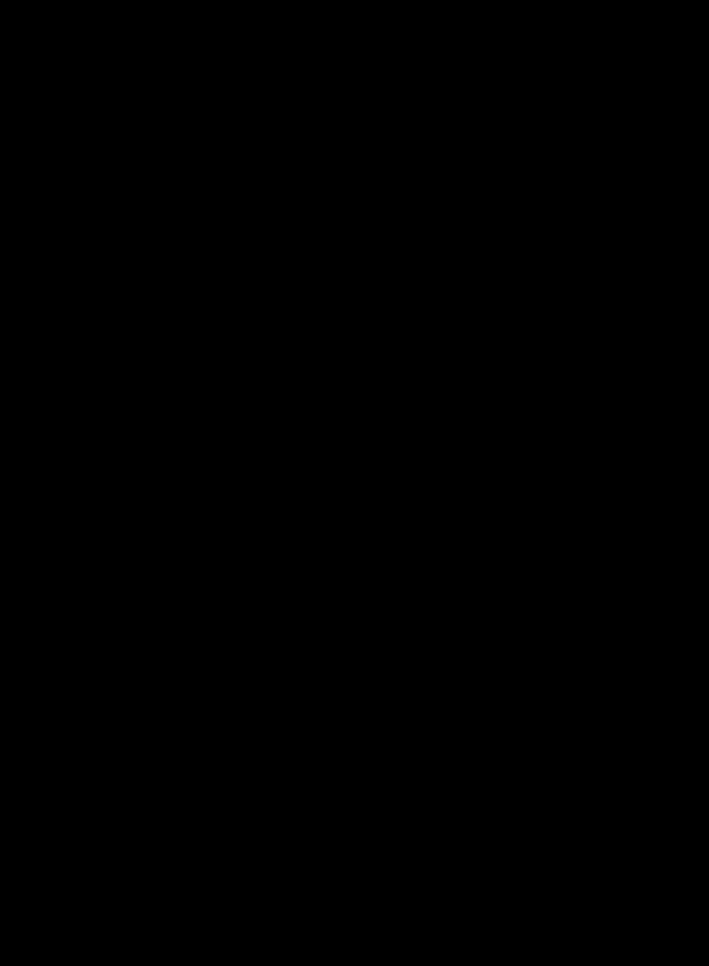
ATP1A3 could be important for CS‐6 treatment efficacy. A‐B, Cell viability assays for testing the suppressive effect of CS‐6, CS‐6, CS‐7, CB and RB (48 h) in U87, U251, LN229 and U118 cells. U118 showed obvious resistance to CS‐6 treatment compared with other cell lines. C, Target fishing experiment for identification of the core proteins targeted by CS‐6. After the SPR experiment, all the target proteins were treated with trypsin after elution from the chip, and HPLC‐MS/MS identification experiments were then performed. D, Western blotting analysis to confirm the ATP1A3 and ATP1A4 expression levels in our panel of GBM cell lines. E, Knocking down ATP1A3 expression by an shRNA‐targeting strategy. F, Confirmation of transfected U87 cells overexpressing ATP1A4 via Western blotting. G, Cell viability assays to examine whether knocking down ATP1A3 or ATP1A4 impacts the anti‐cancer efficacy of CS‐6 (48 h). *P < .05, **P < .01. H, Clonogenic assays testing the anti‐cancer effect of 50 nmol/L CS‐6 (10 h) under sh‐ATP1A3 treatment compared with the sh‐NC treatment. **P < .01. I, Cell cycle analysis exploring whether ATP1A3 impacts the cell cycle distribution changes induced by CS‐6 (24 h). **P < .01. J, Transwell assays examining the differences in anti‐cancer efficacy of CS‐6 (24 h) between the sh‐ATP1A3 treatment group and the sh‐NC group. **P < .01. K, Viability assays of U87 and U251 cells with established downregulation of ATP1A3 expression and control cells treated with 50 μmol/L TMZ, 10 nmol/L CS‐6 or the combination of 50 μmol/L TMZ and 10 nmol/L CS‐6 for 96 h. **P < .01

To further explore the molecular mechanisms underlying the anti‐cancer effect of cardiac glycosides, we analysed the differential inhibitory effects of 5 commonly used compounds from ChanSu on our panel of GBM cells. We found that compared with U87, U251 and LN229 cells, U118 cells showed significantly more resistance to CS‐6 treatment (Figure 2A,B). Intriguingly, for the other 12 clinical agents that were commonly used as anti‐cancer drugs, no significant differences between U87 and U118 cells were observed (Figure S1). We thus further determined the potential mechanisms underlying the differential CS‐6 sensitivity in various GBM cells. For the first time, a target fishing experiment was used to explore the potential targets of CS‐6 in human GBM cells, as well as the differences in potential targets or signalling pathways between CS‐6‐resistant and CS‐6‐sensitive cells (Figure 2C). We found that the target proteins captured by CS‐6 in U87 cells were distinctly different from those captured in U118 cells (Figure 2C). In U87 cells, HPLC‐MS/MS analysis identified 148 target proteins, while in U118 cells, 99 completely different target proteins were found (Table S2). Most compounds from ChanSu are potential sodium pump inhibitors, and here, we identified ATP1A3 as one of the many target proteins captured by CS‐6 in U87 cells and ATP1A4 as a protein in U118 cells. Both ATP1A3 and ATP1A4 are sodium pump subunits in mammals, and thus, we proposed that these two proteins might be involved in the anti‐cancer effects of CS‐6 in GBM cells. Interestingly, ATP1A1 was captured by CS‐6 in GBM cells. However, in contrast to the ATP1A3 results, no obvious difference in the ATP1A1 protein levels between U87 and U118 cells was detected (Figure S1), and more importantly, we confirmed that silencing ATP1A1 in U87 cells did not affect the synergistic therapeutic effect of CS‐6 and TMZ.

We next detected the expression of ATP1A3 and ATP1A4 in various GBM cell lines, and the results indicated that ATP1A3 was generally highly expressed in various cell lines, while it showed nearly no expression in U118 cells (Figure 2D). The expression of ATP1A4 in various GBM cells was generally low, but only high expression levels were detected in U118 cells (Figure 2D). We thus knocked down ATP1A3 by using shRNA (Figure 2E) and overexpressed ATP1A4 in U87 cells (Figure 2F). Intriguingly, the results indicated that U87 cells with ATP1A3 silencing were significantly more resistant to CS‐6 than control cells (Figure 2G, upper panel), while transfected U87 cells overexpressing ATP1A4 showed no detectable changes in the sensitivity to CS‐6 (Figure 2G, lower panel). These findings indicated that ATP1A3 may be an important target protein affecting CS‐6 sensitivity in GBM. In addition, we used a colony formation assay to explore the effect of silencing ATP1A3 on the inhibitory effect of CS‐6 in GBM. The results indicated that suppressing ATP1A3 significantly reversed the inhibitory effect of CS‐6 on GBM cells (Figure 2H). Similarly, using cell cycle analysis, we confirmed that ATP1A3 inhibition attenuated the CS‐6‐mediated blockade of the GBM cell cycle (Figure 2I). Transwell assays also suggested that suppressing ATP1A3 reversed the inhibitory effect of CS‐6 on GBM invasiveness (Figure 2J). Furthermore, the role of SNRPG in the context of mismatch repair (MMR) was analysed, and the outcomes showed that SNRPG inhibition increased the protein expression of the MMR protein MLH1, whose decreased expression could be one of the mechanisms of TMZ resistance (Figure S1). The results indicated that MLH1 expression was significantly increased upon CS‐6 treatment compared to that of the control group, while silencing ATP1A3 reversed the CS‐6‐mediated increases in MLH1. In addition, the MGMT promoter methylation status is important in determining TMZ sensitivity in GBM; thus, the possible correlations between CS‐6 treatment and MGMT methylation status, as well as the effect of ATP1A3 on CS‐6‐induced changes in MGMT methylation status, were explored (Figure S1).

Finally, by using transfected U87 or U251 cells with ATP1A3 knockdown, we further performed cell viability assays to examine the effect of ATP1A3 on the anti‐glioma efficacy of CS‐6, as well as the synergistic therapeutic effect of CS‐6 and TMZ (Figure 2K). Intriguingly, silencing ATP1A3 in U87 and U251 cells increased the synergistic therapeutic effect of CS‐6 and TMZ. In summary, we found that ATP1A3 might be a potential target of CS‐6 in GBM and that regulating ATP1A3 could significantly impact the anti‐glioma efficacy of CS‐6 and its combination with TMZ. More efforts should be directed towards clarifying the roles of ATP1A3 in GBM treatment, and this will promote the clinical use and application of CS‐6.

### 3.3 Validation of ATP1A3‐AQP4 interactors

**Figure 3:**
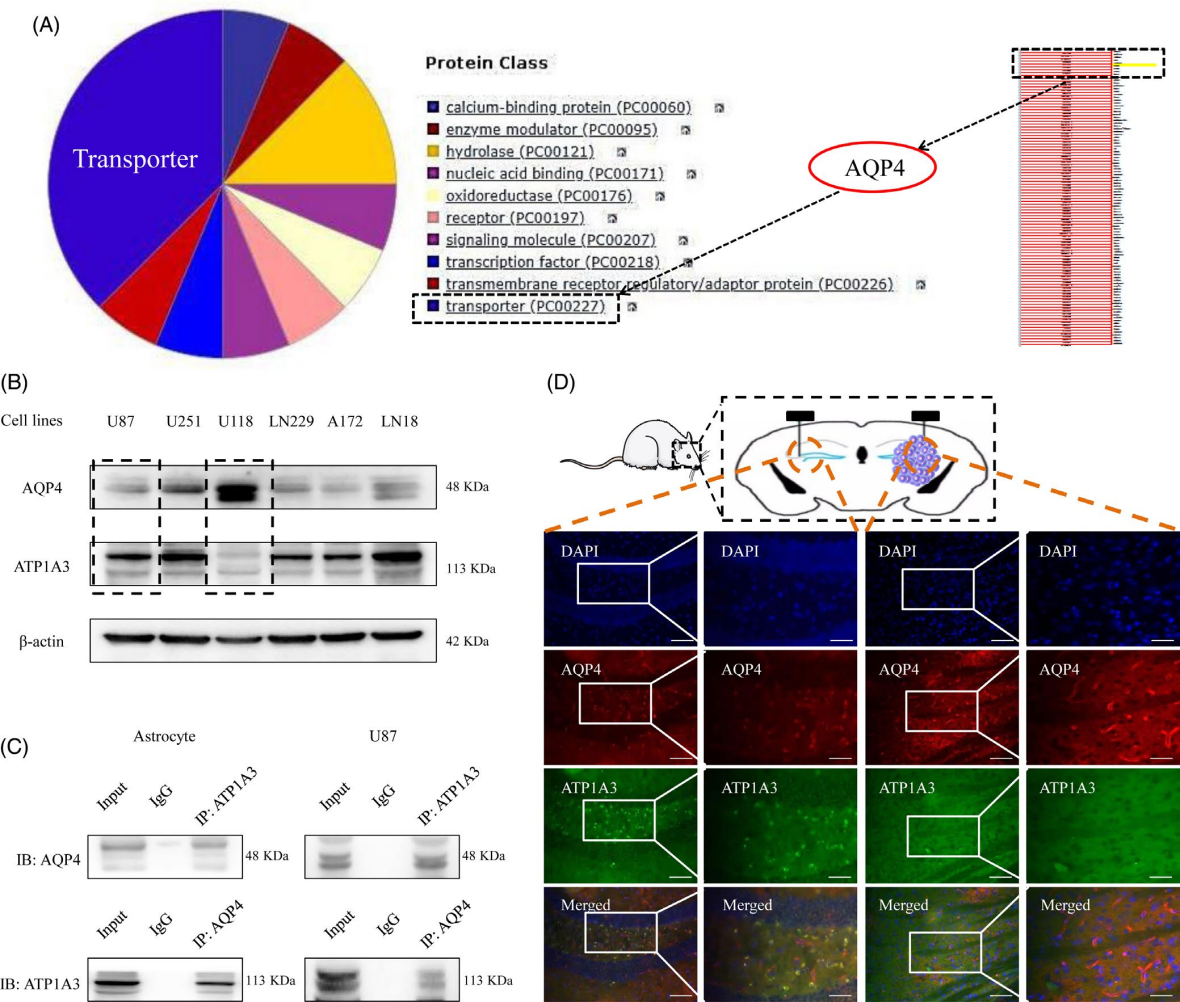
The intimate connection between ATP1A3 and AQP4 in GBM. A, The PANTHER protein classification of the ATP1A3 interactome shows the percentage of each protein class against the total number of proteins with a class hit. The largest class of proteins in the ATP1A3 interactome from whole‐cell lysates are the transporter proteins (left panel). Quantitative MS analyses showed that AQP4 was present in the ATP1A3 complexes (right panel). PC numbers refer to PANTHER class ID. B, AQP4 and ATP1A3 expression levels in various GBM cell lines using Western blotting. C, Colocalization of ATP1A3 and AQP4 was confirmed by immunoblotting analysis, and ATP1A3 was found to directly interact with AQP4 in both normal astrocytes (left panel) and U87 cells (right panel). D, Colocalization of ATP1A3 and AQP4 was confirmed by confocal immunofluorescence analysis using sections of normal brain tissues (left panels) and U87 orthotopic xenografts (right panels). Scale bars, 50/20 μm

To identify the key functional interacting partners of ATP1A3, this study utilized immunoprecipitation‐mass spectrometry (IP‐MS) to identify the potential proteins in whole‐cell lysates of U87 cells, and results showed an enrichment of transporter proteins (Figure 3A, left panel). Quantitative MS analyses showed that AQP4 was present in the ATP1A3 complexes (Figure 3A, right panel). We further analysed ATP1A3 and AQP4 expression in our panel of GBM cells, and intriguingly, we found that ATP1A3 had relatively high expression in U87, U251, LN229, A172 and LN18 cells but was nearly undetectable in U118 cells. AQP4 was only highly expressed in U118 cells and almost undetectable in the other cells (Figure 3B). We next performed Co‐IP to further explore the potential functional interactions between ATP1A3 and AQP4. First, we immunoprecipitated ATP1A3 from U87 cell extracts and detected AQP4 by immunoblotting, and we then immunoprecipitated AQP4 and detected ATP1A3 by immunoblotting. The results indicated that ATP1A3 directly interacts with AQP4 (Figure 3C). To further confirm this interaction, we used normal hippocampal tissues as well as xenografts from the corresponding brain regions in mice to detect the expression levels of these two proteins. Firstly, H&E staining was used for confirming normal brain tissues or orthotopic xenografts (Figure S2). Furthermore, the results of immunofluorescence histochemical staining further indicated that ATP1A3 and AQP4 colocalized in normal brain tissues (left panel), and ATP1A3 was decreased while AQP4 was increased significantly in GBM tissues (right panel) compared with normal tissues (Figure 3D), suggesting that the expression of ATP1A3 might be negatively correlated with AQP4 expression. Therefore, in the following text, we further explored the molecular mechanisms underlying their synergistic effects in regulating malignant growth of GBM. We expected that the functional interaction between ATP1A3 and AQP4 might mediate the anti‐cancer effect of CS‐6 and the chemosensitizing effect of CS‐6 on TMZ.

### 3.4 Inhibition of AQP4 sensitizes GBM cells to TMZ through the post‐transcriptional regulation of p38

**Figure 4:**
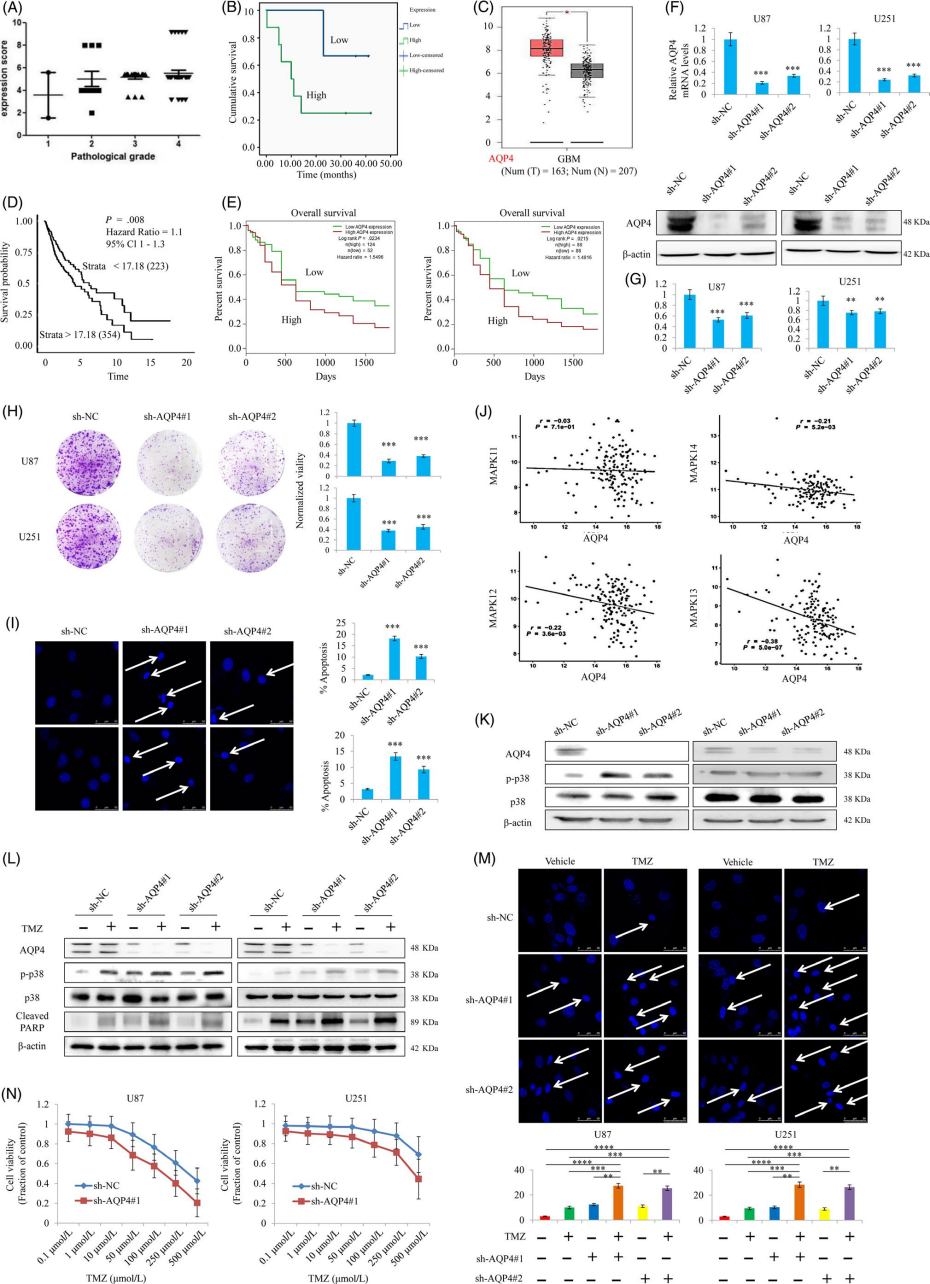
AQP4 suppression promotes TMZ chemosensitivity in GBM. A, Clinical data of ATP1A1 expression levels in human tissue microarrays from glioma patients. B, Kaplan‐Meier analysis of overall survival (OS) for GBM patients with different expression levels of AQP4 by a log‐rank test. “Low” indicates a low level of AQP4 expression; “high” indicates a high level of AQP4 expression. C, The correlation of AQP4 RNA levels with the incidence of GBM was examined with R programming language. A total of 163 normal brain samples and 207 GBM samples were selected for analysis. We then performed a t test using R programming language with the criteria of |log2FC| >1 and *P* value <.05. D, With R programming language, 207 GBM samples were selected and analysed to explore the correlation between AQP4 expression levels and the corresponding patient survival data (*P* < .01). E, Rembrandt data from TCGA were downloaded, and R programming language was used to perform survival analysis (*P* < .05). F, AQP4 mRNA expression levels in GBM were determined by quantitative RT‐PCR analysis (upper panels). β‐Actin was used as the internal control. AQP4 protein abundance in the GBM cells, as indicated, was determined by Western blotting analysis (lower panels). G, Cell viability measured in transfected U87 and U251 cells with downregulation of AQP4 for three days. Note: sh‐AQP4#1 targets a specific sequence in the 3'‐UTR of AQP4; sh‐AQP4#2 targets a specific sequence in the open reading frame of AQP4. H, GBM cells transfected with sh‐AQP4 or sh‐NC were cultured for 14 d and were then stained with crystal violet. I, Apoptosis was quantified by DAPI staining as well as Annexin V assay of sh‐NC‐ or sh‐AQP4‐transfected GBM cells, as indicated. Scale bar, 50 μm. ***P < .001. J, In TCGA data, the correlation between AQP4 expression and p38 (MAPK11‐14) was revealed, suggesting that p38 might be the downstream factor regulated by AQP4. K, The protein abundance of AQP4 and p‐p38 was determined by Western blotting analysis in sh‐NC or sh‐AQP4 GBM cells. L‐M, AQP4 knockdown induces apoptosis in GBM cells upon TMZ treatment. L, The sh‐NC or sh‐AQP4 GBM cells were treated with TMZ and were then subjected to immunoblotting analysis. M, Apoptosis was quantified by DAPI staining (upper panel) as well as Annexin V assay (lower panel), of sh‐NC or sh‐AQP4 GBM cells. The error bars represent the mean ± SD Scale bar, 50 μm. **P < .01, ***P < .001, ****P < .0001. N, Cell viability was measured using sh‐NC or sh‐AQP4 GBM cells treated with TMZ for 72 h. The error bars represent the mean ± SD ***P < .001 vs the sh‐NC group, ^###^ *P* < .0001 vs the TMZ treatment group

AQP4 plays an important role in the anti‐glioma effect of TMZ; however, the exact mechanisms still need more research. For the first time, we used a human brain tissue microarray to detect the AQP4 protein expression in different grades of human GBM, and the results indicated that the AQP4 protein levels were strongly associated with the degree of malignancy in glioma (Figure 4A), supporting the important roles of AQP4 in GBM malignancy. Survival analysis also indicated that the prognosis of patients with relatively lower expression of AQP4 was generally better than that of patients with higher expression (*P* < .05) (Figure 4B). In addition, TCGA data analysis indicated that the expression of AQP4 in tumour tissue samples was significantly higher than that in paracancerous tissues (*P* < .05) (Figure 4C), and the overall survival of GBM patients with lower expression of AQP4 was significantly better than that of patients with relatively higher expression (*P* < .01) (Figure 4D). Furthermore, with TCGA, we used Rembrandt data to perform Kaplan‐Meier analysis, and the results indicated that GBM patients with lower AQP4 expression levels had a better prognosis than those with higher AQP4 expression levels (*P* < .05) (Figure 4E).

We next examined the oncogenic ability of AQP4 in GBM. We first knocked down AQP4 by using an shRNA strategy in U87 and U251 cell lines (Figure 4F). Cell viability assays confirmed that AQP4 suppression significantly inhibited GBM cell proliferation after 3 days and 14 days (Figure 4G‐H). To further examine the effect of AQP4 on cell apoptosis, we performed a cell apoptosis assay, and the results indicated that silencing AQP4 significantly promoted the GBM cell apoptosis rates, via both immunofluorescence assay (Figure 4I, left panel) and Annexin V assay (Figure 4I, right panel). In addition, by analysing the TCGA database, we found a negative correlation between AQP4 and all p38‐encoding genes (MAPK11‐14) (*P* < .01) (Figure 4J). AQP4 therefore likely regulates the p38‐MAPK signalling pathway. Then, we silenced AQP4 by using shRNA and found that AQP4 suppression significantly promoted p38 phosphorylation (Figure 4K). Further immunoblotting analysis indicated that TMZ or sh‐AQP4 alone resulted in only a slight increase in the phosphorylation level of p38; however, the combination of AQP4 knockdown and TMZ induced a substantial increase in p38 phosphorylation (Figure 4L). Similarly, TMZ or sh‐AQP4 alone resulted in only a moderate increase in the apoptotic signal, as determined by Western blotting analysis of cleaved PARP protein abundance. The combination of AQP4 knockdown and TMZ induced substantial apoptosis (Figure 4L‐M). In addition, the cell viability assay confirmed that silencing AQP4 significantly increased the TMZ sensitivity of GBM cells (Figure 4N). These results indicated that AQP4 blockade sensitized GBM cells to TMZ.

### 3.5 CS‐6 induces a negative feedback loop connecting ATP1A3 expression and the AQP4 pathway

**Figure 5:**
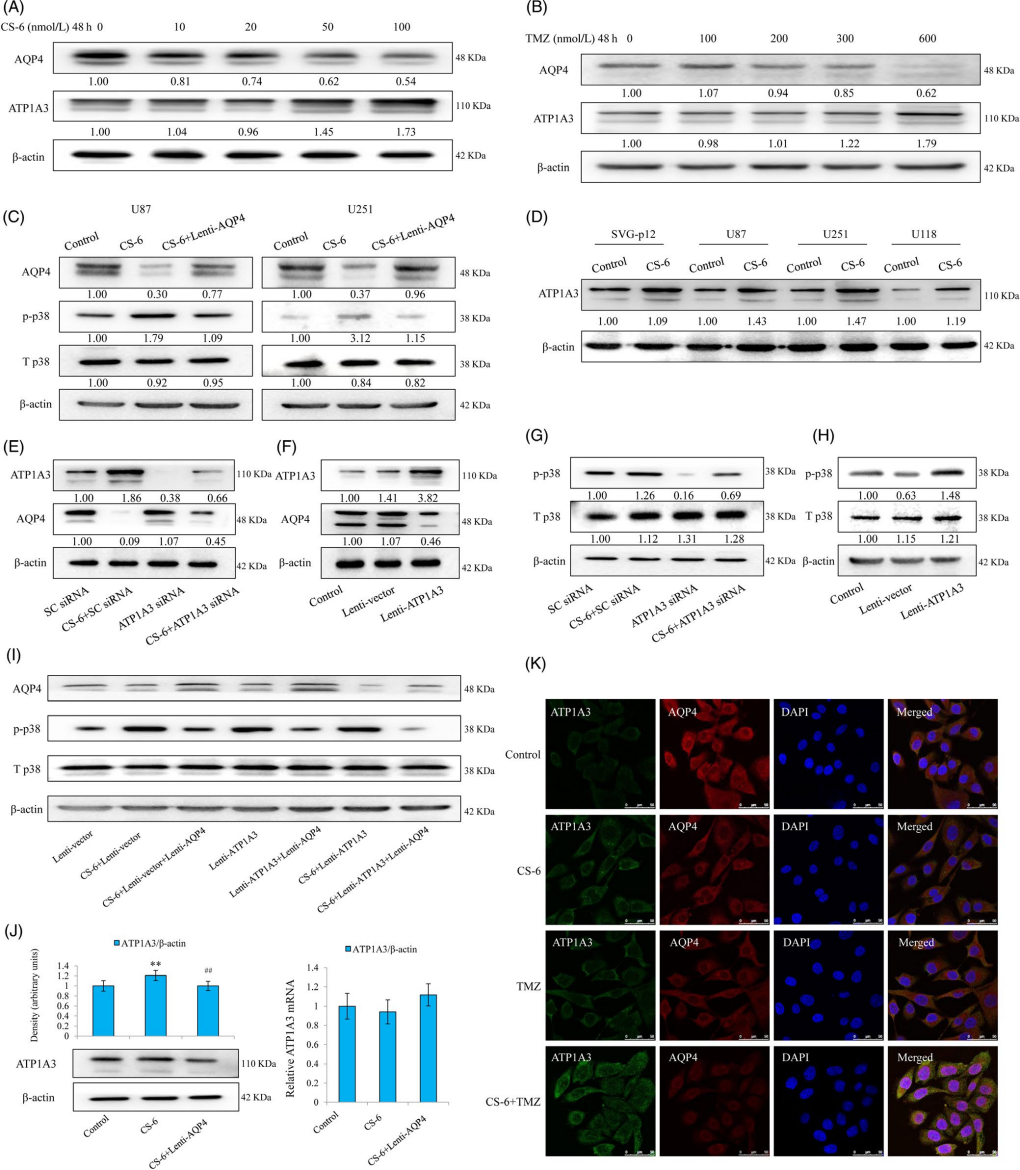
CS‐6 activates the negative feedback loop connecting ATP1A3 expression and the AQP4 pathway. A, ATP1A3 and AQP4 expression changes induced by CS‐6 in U87 cells. B, ATP1A3 and AQP4 expression changes induced by TMZ in U87 cells. C, CS‐6 induces hyperphosphorylation of the p38 protein in an AQP4‐dependent manner. Western blotting analysis indicated the levels of AQP4 and p‐p38 in GBM cells exposed to CS‐6 (50 nmol/L) treatment or CS‐6 treatment plus AQP4 overexpression for 4 d. **P < .01 versus the control group; ^##^ *P* < .01 vs the CS‐6 treatment group. Error bars represent the standard error of the mean. D‐F, CS‐6‐induced ATP1A3 overexpression regulates AQP4 activation. D, Western blotting showed the significant expression changes of ATP1A3 in GBM cells exposed to 50 nmol/L CS‐6 for 3 d. However, the results in normal embryonic SVG‐p12 astrocytes did not show similar changes. E, Knockdown of ATP1A3 reversed the CS‐6‐induced upregulation of ATP1A3 and downregulation of AQP4 in U87 GBM cells. **P < .01 versus the control group; ^##^ *P* < .01 versus the CS‐6 (50 nmol/L) treatment group. F, Overexpression of ATP1A3 downregulated the levels of AQP4 in U87 GBM cells. **P < .01 versus the control group. G‐I, ATP1A3 regulates CS‐6‐induced hyperphosphorylation of the p38 protein via regulating the AQP4 signalling pathway in U87 GBM cells. G, CS‐6‐induced p38 hyperphosphorylation via ATP1A3. **P < .01 vs SC siRNA; ^##^ *P* < .01 vs the CS‐6 (50 nM) treatment group. H, Overexpression of ATP1A3 promoted the hyperphosphorylation of the p38 protein. **P < .01 vs the lenti‐vector group. (I) The AQP4 signalling pathway is required for ATP1A3/CS‐6‐induced p38 hyperphosphorylation. **P < .01 vs the control group; ^#^ *P* < .05, ^##^ *P* < .01 and ^###^ *P* < .001 vs the indicated groups. J, CS‐6 increases ATP1A3 expression in an AQP4‐dependent manner in U87 cells. Western blotting (left panel) and real‐time quantitative RT‐PCR (right panel) indicated that CS‐6 (50 nmol/L) upregulated the expression of ATP1A3 in an AQP4‐dependent manner at the post‐transcriptional level. K, The protein expression changes of ATP1A3 and AQP4 upon CS‐6/TMZ monotherapy or the addition of CS‐6 to TMZ in U87 cells, determined by using confocal immunofluorescence. **P < .01 vs the control group; ^##^ *P* < .01 vs the CS‐6 (50 nM) treatment group

First, we found that both TMZ and CS‐6 significantly increased the expression of ATP1A3 (Figure 5A,B) and together decreased AQP4 expression (Figure 5A,B). Then, we further examined the effect of CS‐6, as well as AQP4 regulation, on the phosphorylation of p38. The results showed that CS‐6 significantly induced the hyperphosphorylation of p38, while AQP4 overexpression hindered the occurrence of this induction (Figure 5C). Based on these results, we concluded that CS‐6 induces hyperphosphorylation of p38 protein in an AQP4‐dependent manner.

Next, we explored whether ATP1A3 mediates AQP4 suppression upon CS‐6 treatment. We first examined the effect of CS‐6 on ATP1A3 expression in our panel of GBM cell lines, and we found that in U87, U251 and U118 cells, CS‐6 significantly increased ATP1A3 expression, while this effect was not as obvious in human normal SVG‐p12 cells as in cancer cells (Figure 5D). Next, we further explored the effect of ATP1A3 on CS‐6‐induced AQP4 suppression. Western blotting analysis indicated that CS‐6 significantly suppressed AQP4 expression, but silencing ATP1A3 reversed the decrease in AQP4 expression induced by CS‐6 (Figure 5E). In addition, ATP1A3 overexpression significantly blocked AQP4 expression (Figure 5F). Thus, we concluded that CS‐6‐induced ATP1A3 overexpression regulates AQP4 activation.

We next examined the effect of CS‐6 on the phosphorylation of p38 and investigated whether ATP1A3 could mediate the changes in p38 phosphorylation upon CS‐6 treatment. Our results indicated that CS‐6 significantly upregulated p38 phosphorylation and that silencing ATP1A3 attenuated the hyperphosphorylation induced by CS‐6 (Figure 5G). Meanwhile, ATP1A3 overexpression induced the hyperphosphorylation of p38 (Figure 5H). Thus, we concluded that CS‐6 impacts p38 phosphorylation by regulating ATP1A3. Interestingly, AQP4 overexpression attenuated p38 hyperphosphorylation induced by CS‐6 treatment or ATP1A3 overexpression (Figure 5I). Thus, ATP1A3 regulates CS‐6‐induced hyperphosphorylation of the p38 protein via the AQP4 signalling pathway.

Then, we further explored the potential regulatory effect of AQP4 in the CS‐6‐induced increase in ATP1A3. Immunoblotting analysis indicated that overexpression of AQP4 significantly reversed CS‐6‐mediated ATP1A3 activation (Figure 5J). However, overexpression of AQP4 failed to alter ATP1A3 mRNA expression upon CS‐6 treatment. Thus, we concluded from this result that CS‐6 increases ATP1A3 expression in an AQP4‐dependent manner.

In addition, we used CS‐6/TMZ monotherapy or the addition of CS‐6 to TMZ to examine the protein expression changes of ATP1A3 and AQP4 by confocal immunofluorescence. The results indicated that ATP1A3 and AQP4 colocalized in GBM cells, and both CS‐6 and TMZ monotherapy promoted the activation of ATP1A3 and, together, AQP4 suppression. Importantly, compared with monotherapy, combined TMZ and CS‐6 treatment induced more significant expression changes of ATP1A3 and AQP4 (Figure 5K). Thus, CS‐6 induces a negative feedback loop connecting ATP1A3 expression and the AQP4 pathway to aggravate the formation of p38 hyperphosphorylation in GBM cells, which might be potentially important for the synergistic treatment effect of CS‐6 and TMZ.

### 3.6 CS‐6‐induced activation of human ATP1A3 requires Thr794

**Figure 6:**
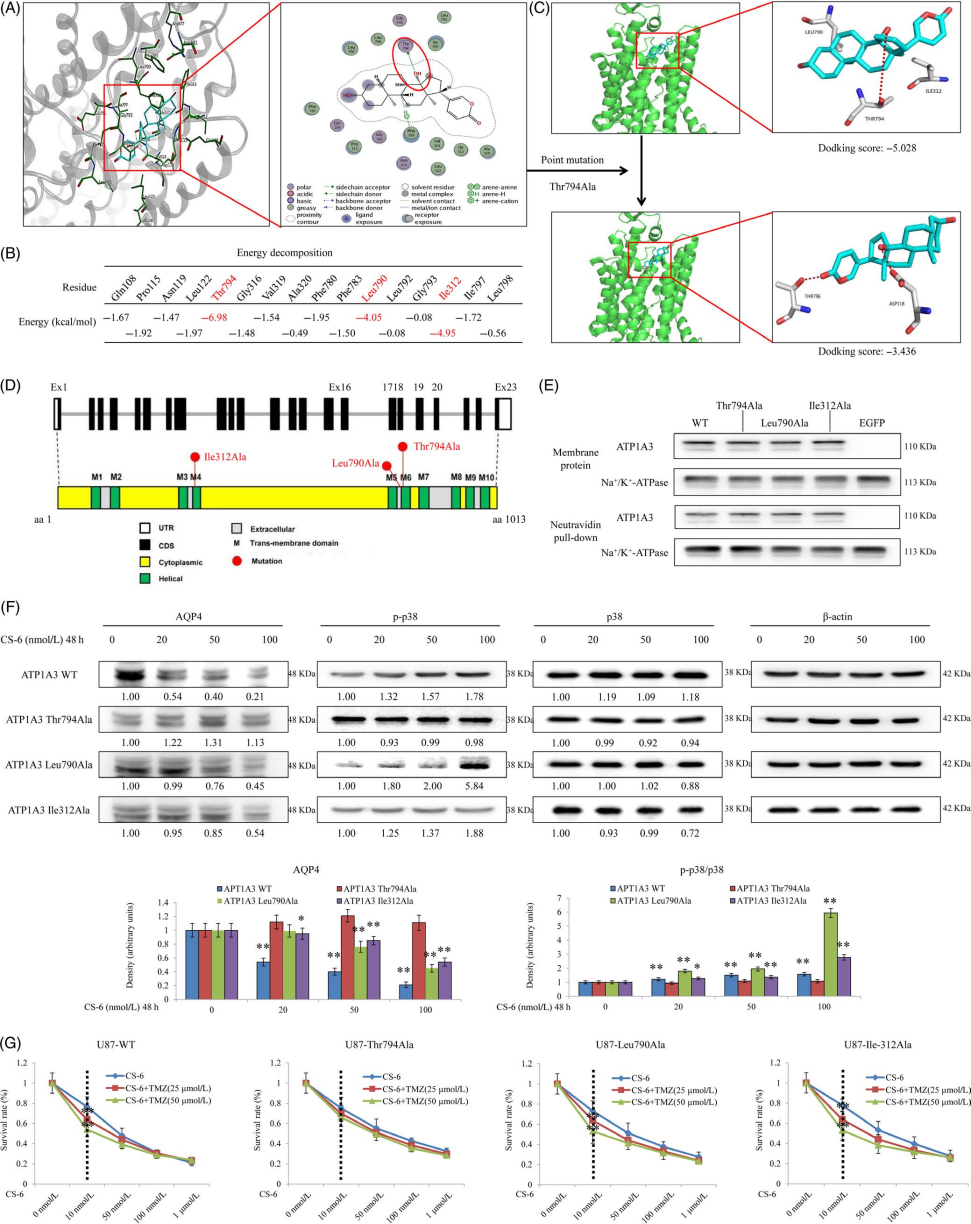
Abrogating hydrogen bonding of the amino acid Thr794 interferes with activation of ATP1A3 by CS‐6. A, Docking conformations of CS‐6 and ATP1A3 by using Molecular Operating Environment. Interactions between CS‐6 and the key residues of ATP1A3 in 3D (left panel) and 2D (right panel) models with low binding energy are shown. H‐bond interactions between CS‐6 and the active site residues (Thr794) of ATP1A3 formed. B, Binding free energy scoring for the top 16 poses of CS‐6. C, The details of the predicted binding mode of CS‐6 and wild‐type ATP1A3 protein (upper panel) or ATP1A3 protein with the Thr794Ala mutation (lower panel) using Schrödinger. The contact residues are shown and labelled by type and number. The red dotted line illustrates the hydrogen bond interaction. The docking score for wild‐type ATP1A3 was −5.028, while the score for ATP1A3 with the Thr794Ala mutation was −3.436, indicating that the binding strength between mutated ATP1A3 and CS‐6 was significantly weaker than that between wild‐type ATP1A3 and CS‐6. D, Diagram of three site‐directed mutations in the ATP1A3 protein. E, The point mutations at Thr794, Leu790 and Ile312 do not interfere with ATP1A3 expression in the plasma membrane. U87 cells were transiently transfected with an expression vector for EGFP and either wild‐type ATP1A3 or ATP1A3 carrying the indicated mutation. After transfection for 48 h, cells were surface‐biotinylated, followed by isolation of membrane proteins. Membrane protein (400 mg) was incubated with NeutrAvidin agarose, and biotinylated proteins were isolated. On one 7.5% SDS‐PAGE gel, total membrane protein (10 mg) for each condition was separated, and on another 7.5% SDS‐PAGE gel, the complete supernatant from the pulldown for each condition was separated. Both were transferred onto a PVDF membrane and split in half between the marker bands for ATP1A3 and Na^+^/K^+^‐ATPase. Each part was probed for the corresponding protein. All mutant forms of ATP1A3 could be detected in the plasma membrane. F, Impact of the mutations on the CS‐6‐induced activation of downstream signalling, including AQP4 expression and p38 phosphorylation in U87 cells. *P < .05 and **P < .01 versus the control group. G, Impact of the mutations on the synergistic treatment effect of CS‐6 and TMZ in our panel of GBM cell lines. Cells were transfected with an expression vector for either wild‐type ATP1A3 or ATP1A3 carrying the indicated mutations. After transfection for 24 h, cells were treated with CS‐6 or the addition of different concentrations of CS‐6 to 25 μmol/L/50 μmol/L TMZ for 48 h and subjected to a cell viability assay for U87‐WT (IC_50_ values: 0.047, 0.035 and 0.011 μmol/L for three groups, respectively), as well as different mutations, including U87‐Thr794Ala (IC_50_ values: 0.068, 0.062 and 0.059 μmol/L for three groups, respectively), U87‐Leu790Ala (IC_50_ values: 0.059, 0.045 and 0.029 μmol/L for three groups, respectively) and U87‐Ile312Ala (IC_50_ values: 0.058, 0.036 and 0.019 μmol/L for three groups, respectively)

To further explore the potential mechanisms underlying CS‐6‐mediated promotion of ATP1A3, we performed homology modelling, as well as protein‐ligand docking. To validate the quality of the constructed model, we generated the Ramachandran plot for the modelled structure as shown in Figure S3. All residues are located in the favoured or allowed regions, and no outliers can be observed. This result indicates the high quality of our model. Docking simulations were conducted to predict the binding mode and binding affinity of the ligand CS‐6 with the receptor ATP1A3. As indicated by the binding modes, the ligand CS‐6 binds strongly to the receptor, mainly through hydrogen bonds and hydrophobic interactions. CS‐6 forms a hydrogen bond with the side chain of Thr794 and forms a CH‐Pi interaction with Phe780 (Figure 6A). The docking process retained 16 top‐scored poses for further analysis. The binding free energy scores of the 16 poses are listed in Figure 5B. From the results, we found that Thr794, Leu790 and Ile312 were the three most likely binding sites with the most optimal energy configurations (Figure 6B). Combining visual inspection and empirical analysis, we selected one most representative pose for the ligand CS‐6, whose 3D and 2D binding modes are depicted in Figure 6A.

Next, we examined whether abolishing the hydrogen bonding or other interactions from certain amino acid residues interferes with activation of ATP1A3 by CS‐6. Examination of the homology model with the various docked and modelled poses indicated that the amino acid residue Thr794 will be in proximity to the ligand and therefore may interact with CS‐6. To determine the role of this amino acid residue, we performed a computer simulation to investigate how specific point mutations impact molecular binding. We further performed molecular docking analysis by using Schrödinger analysis, and the details of the predicted binding mode of CS‐6 and wild‐type ATP1A3 protein (upper panel) or ATP1A3 protein with the Thr794Ala mutation (lower panel) are shown in Figure 6C,D. The docking score for wild‐type ATP1A3 was −5.028, while the score for ATP1A3 with the Thr794Ala mutation was −3.436. These results suggested that the binding between wild‐type ATP1A3 and CS‐6 was significantly stronger than that between mutated ATP1A3 and CS‐6. In addition, docking analysis between CS‐6 and ATP1A3 with the Ile312Ala or Leu790Ala mutation was also performed, and similar results were found (Figure S4), indicating that these three point mutations—Thr794Ala, Leu790Ala and Ile312Ala—could significantly hamper the binding of CS‐6 and the ATP1A3 protein.

Furthermore, we introduced point mutations experimentally at various positions in the ATP1A3 expression vector. While the mutations were deliberately chosen to be conservative, we could not exclude misfolding, degradation or lack of trafficking of the channel to the plasma membrane. We therefore assessed expression at the cell surface in transiently transfected cells by biotinylation and pulldown of surface proteins. As shown in Figure 6E, all of the mutants were detected in the plasma membrane of the transfected cells, while the channel was not detected in the membranes of cells transfected with the vector backbone (pIRES2‐EGFP).

To investigate the effect of the point mutations on ATP1A3, we further examined whether these mutations affected AQP4 expression and p38 phosphorylation, which could be important for TMZ sensitivity in GBM cells. Immunoblotting analysis indicated that the mutations Leu790Ala and Ile312Ala did not inhibit the regulatory effect of CS‐6 on AQP4 protein expression or p38 phosphorylation. However, GBM cells transfected with the Thr790Ala mutant protein showed different protein expression patterns after treatment with CS‐6, and both the decreased AQP4 and elevated p‐p38 expression induced by CS‐6 could be abolished by the Thr790Ala mutation (Figure 6F). Interestingly, a cell viability assay also indicated that the Thr790Ala mutation abolished the synergistic effect of CS‐6 and TMZ in treating GBM (Figure 6G). Overall, our complementary results using point mutations to abrogate individual hydrogen bonds suggested by a homology model of human ATP1A3 support the proposed CS‐6‐binding mode, provide insights into the critical residues for ATP1A3 channel activation and, more importantly, identify mechanisms clarifying how the negative feedback loop connecting ATP1A3 expression and the AQP4‐p38 pathway influences the synergistic treatment effect of CS‐6 and TMZ in GBM cells.

### 3.7 Synergistic effects of CS‐6 and TMZ in GBM in vivo

**Figure 7:**
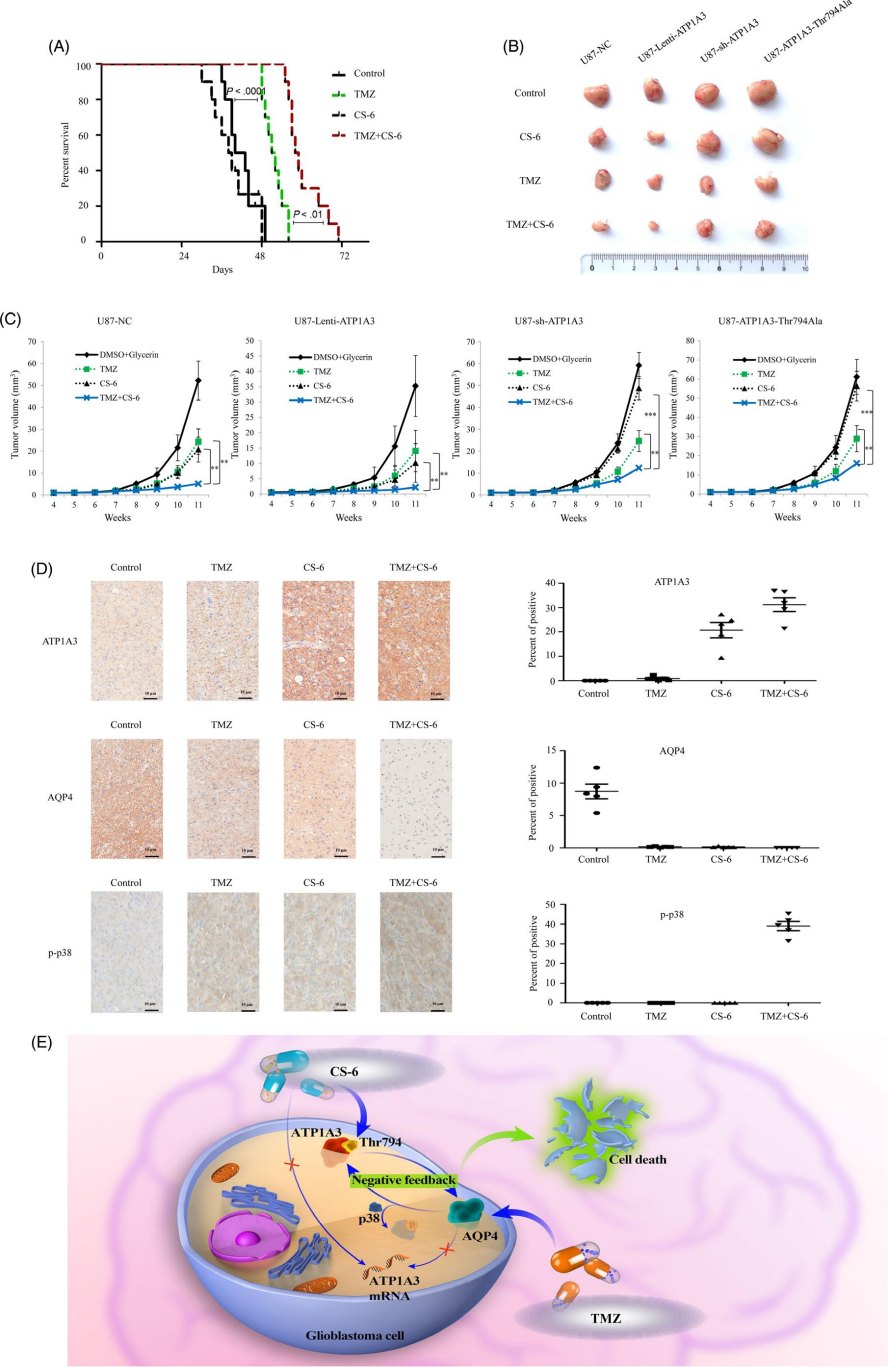
TMZ in combination with CS‐6 significantly inhibits the growth of GBM xenografts. A, Kaplan‐Meier survival curves indicating prolonged survival of mice with subcutaneous U87 xenografts after treatment with the combination of CS‐6 and TMZ compared to monochemotherapy. B‐C, GBM tumour growth at week 11 in animals with U87 xenografts treated with control vehicle, CS‐6, TMZ or the combination of CS‐6 and TMZ. Representative tumour tissues (B) and growth curves (C) demonstrated a significantly reduced tumour burden in animals receiving CS‐6 plus TMZ compared to TMZ or CS‐6 alone. D, ATP1A3 and AQP4 expression in tumours was determined by IHC assays (scale bar, 10 μm). Quantitative analysis was performed using the IHC profiler in ImageJ. Two‐way analysis of variance (ANOVA) followed by Bonferroni's test for multiple comparisons was performed to analyse the data. n = 5. Statistical differences are given as **P < .01 and ***P < .001. E, Suggested mechanisms of the synergistic effect of CS‐6 and TMZ against GBM. The CS‐6‐induced negative feedback loop involving ATP1A3 expression and AQP4 pathway aggravates the formation of p38 hyperphosphorylation and subsequently results in sensitization to TMZ chemotherapy, which strongly relies on the amino acid Thr794 in ATP1A3. We suggest that CS‐6, by activating the feedback loop between ATP1A3 and AQP4, improves TMZ cytotoxicity

The therapeutic effect of combined CS‐6 and TMZ was further explored in vivo in mice carrying subcutaneously implanted U87 xenografts. After implantation for 10 days, mice were randomly divided into 4 groups: control (DMSO + glycerine) (n = 5), CS‐6 (1 mg/kg; n = 5), TMZ (20 mg/kg; n = 5) and CS‐6 plus TMZ (1 mg/kg + 20 mg/kg; n = 5). Survival analysis indicated that CS‐6 monotherapy did not improve the prognosis of the mice with GBM, while TMZ alone significantly prolonged the survival time of the mice (Figure 7A). Intriguingly, the prognosis of combined CS‐6 and TMZ treatment was obviously better than that of TMZ monotherapy. This finding further confirmed our previous results supporting the chemosensitizing effect of CS‐6. Meanwhile, by analysing the tumour volumes and tumour growth curves, we found that the treatment effect of CS‐6/TMZ monotherapy was not as strong as that observed by combining CS‐6 and TMZ (Figure 7A,B), indicating that the CS‐6 and TMZ cotreatment significantly extended overall survival compared to TMZ treatment alone. In addition, we found that increased expression of ATP1A3 promoted the synergistic effect of CS‐6 and TMZ, while silencing ATP1A3 or the ATP1A3 point mutation Thr794Ala blocked this synergistic treatment effect (Figure 7C). Overall, ATP1A3 could be important for the anti‐cancer effect as well as the chemosensitizing effect of CS‐6. However, ATP1A3 did not impact the treatment efficacy of TMZ monotherapy. Furthermore, IHC analysis indicated that ATP1A3 increased and AQP4 decreased following treatment with CS‐6/TMZ monotherapy or cotreatment with CS‐6 and TMZ (Figure 7D). H&E staining demonstrating the features of the tumours in different group has also been exerted (Figure S5). These results further showed in vivo that both ATP1A3 and AQP4 could have value for the synergistic effect of CS‐6 and TMZ cotreatment in GBM.

In summary, our findings confirmed that CS‐6 acts as a potent sensitizer for TMZ chemotherapy in vitro and in vivo with the potential to reduce the tumour burden and prolong survival. Our results unequivocally demonstrated that CS‐6‐induced ATP1A3 overexpression inhibits AQP4, which, in turn, causes an increase in ATP1A3 expression at the post‐transcriptional level, resulting in sustained suppression of AQP4 and hyperphosphorylation of p38. This finding suggests that CS‐6 triggers a negative feedback loop involving ATP1A3 and AQP4 to amplify the formation of p38 hyperphosphorylation in GBM cells (Figure 7E). Therefore, strategies to activate the feedback loop may prove beneficial for suppressing GBM growth and help provide an experimental basis for further assessment of combined TMZ and CS‐6 treatment in an adjuvant clinical setting.

## 4 DISCUSSION

CS‐6 has been widely used in anti‐cancer experimental research, but its cellular toxicity limits its further clinical application. Thus, reducing the toxicity of CS‐6 to normal cells or enhancing the anti‐cancer efficacy at low concentrations is currently a major goal. Based on our previous research, we further explored the anti‐glioma effect of CS‐6, as well as the practicability of its combination with TMZ in treating GBM. As the most commonly used clinical anti‐glioma agent, TMZ has the issues of increased resistance and various side effects for GBM patients.^28^, ^29^ Thus, an auxiliary drug that can enhance the therapeutic effect of TMZ or reduce the dosage of TMZ will substantially improve the prognosis of GBM patients. In the current study, we further explored the anti‐glioma mechanisms of CS‐6 and, more importantly, clarified its chemosensitizing effect on TMZ, which will provide a theoretical basis for future clinical research on CS‐6. The current research indicated for the first time that ATP1A3 might exert an important effect on the anti‐cancer activity and chemosensitizing effect of CS‐6, and CS‐6 could be a potent sensitizer of TMZ chemotherapy in GBM via regulating the ATP1A3‐AQP4 signalling pathway.

Druggable target identification is extremely important for identifying therapeutic drugs.^30^ For the first time, a target fishing experiment was used to identify the potential target proteins of CS‐6 in GBM cells. However, many target proteins were captured in the current study, and other undetected proteins might also exert an effect on the anti‐cancer activity of CS‐6. Thus, further optimizing this experimental technology to reduce the number of captured target proteins has become a challenge, and addressing this issue will increase the efficiency of target identification for other kinds of anti‐cancer drugs. Unfortunately, we could not conclude that ATP1A3 is the only target of CS‐6 in GBM because other captured proteins might also be involved in the anti‐cancer activity of CS‐6. More studies should examine this issue, and we will address this problem in future research.

Ion channels and transporters are recurrently mutated in certain cancers.^31^, ^32^ These data suggest that ion channel and transporter mutations can be drivers in certain contexts. For the first time, we identified the importance and potential mechanisms of ATP1A3 in GBM under the treatment of bufadienolide. Mechanistic research further indicated that point mutations in ATP1A3 could exert decisive effects on the anti‐cancer activity and the chemosensitizing effect of CS‐6. ATP1A3 mutations are associated with various CNS diseases, as previously mentioned.^15^, ^16^, ^17^ However, the roles of ATP1A3 mutations in GBM have not been clarified before. Using a homology model with different docked and modelled poses of CS‐6 in the binding pocket, we identified Thr794, Leu790 and Ile312 as amino acids in proximity to the CS‐6 that might potentially engage in hydrogen bonding or otherwise interact with the natural ligand. Further biochemical experiments indicate that the amino acid Thr794 is most likely involved in the molecular interaction between ATP1A3 and CS‐6n and, more importantly, is involved in the chemosensitizing effect of CS‐6 in GBM therapy with TMZ. In this study, because Ala is the simplest of all amino acids, we chose to mutate certain amino acids into Ala to inhibit their functions to the maximum extent, and the subsequent biochemical tests confirmed the residues crucial for ATP1A3 activity. Overall, our study indicated that functional loss of ATP1A3, induced by the point mutation Thr794Ala, could inhibit the anti‐glioma effect of CS‐6, as well as its chemosensitizing effect for TMZ. The current study can also provide new ideas or strategies for mechanistic research of other cancers. In the future, new techniques, such as Ala‐scanning,^33^ could be further used to determine the active sites on the ATP1A3 protein to accelerate the discovery of various other anti‐cancer drugs.

As indicated in the current research, p38‐MAPK may play important roles in the therapeutic resistance of GBM. The p38‐MAPK pathway has been reported to exert important effects on the cellular response to various types of stress, including cancer.^34^ For instance, MAPK13 and MAPK14 are p38 isoforms. MAPK13 encodes p38δ, and epigenetic silencing of MAPK13 has been found to contribute to cancer progression,^35^ while MAPK14 encodes p38α/MAPK, which can inhibit carcinogenesis through suppressing the accumulation of reactive oxygen species (ROS).^36^ Activating the MAPK14 pathway could inhibit cancer cell growth via ROS in cancer.^37^ Interestingly, previous research has also suggested that the anti‐cancer effect of TMZ on GBM could be enhanced by MAPK13‐ and MAPK14‐mediated ROS accumulation.^38^ Currently, the effect of p38‐MAPK on the sensitivity of GBM cells to TMZ treatment and the potential mechanisms still need further research.

In addition, AQP4 has long been studied in various brain pathological conditions, and AQP4 knockout could play important roles in neurodegenerative diseases.^39^ Interestingly, AQP4 deficiency was previously found to cause the dysregulation of Na^+^/K^+^‐ATPase.^40^ The current study comprehensively and systematically explored the therapeutic potential of AQP4 in GBM, and for the first time, we found that AQP4 suppression could significantly promote TMZ sensitivity. By regulating ATP1A3, as well as forming a negative feedback loop connecting ATP1A3, AQP4 exerts important effects in determining the therapeutic efficacy of CS‐6 and TMZ cotreatment. The effect of AQP4 on TMZ sensitivity found in this study could be important for improving GBM treatment because selective inhibition of AQP4 may become a new strategy and research direction for GBM therapy. However, to date, no specific therapeutic agents have been developed to inhibit or enhance AQP4. Our experimental results strongly emphasize the importance of this topic for future investigations.

In conclusion, the current research indicated that CS‐6 could be a potent sensitizer of TMZ chemotherapy in GBM, and combined TMZ and CS‐6 treatment showed a better effect than monotherapy both in vitro and in vivo. CS‐6 might mediate the inhibition of GBM via regulating the ATP1A3‐AQP4 signalling pathway. Furthermore, our complementary results using point mutations to abrogate individual hydrogen bonds proposed by a homology model of human ATP1A3 could provide insights into the critical residue Thr794 for ATP1A3 activation and the synergistic effect of CS‐6 and TMZ. CS‐6 treatment together with TMZ could become a new strategy for treating GBM.

## CONFLICT OF INTERESTS

The authors declare no conflict of interest.

## AUTHOR CONTRIBUTIONS

Y.‐LL and BZ conceived and designed the experiments; Y.‐LL, CC, XW, J.‐CL, J.‐SX, SZ, J.‐L. H. and WL analysed the data; Y.‐LL, CC and XW contributed samples/analysis tools; and Y.‐LL, WL and BZ wrote the paper.

## Data Availability

The data that support the findings of this study are available from the corresponding author upon reasonable request. Research data are not shared.
