# Supplementary material for: Gamabufotalin induces a negative feedback loop connecting ATP1A3 expression and the AQP4 pathway to promote temozolomide sensitivity in glioblastoma cells by targeting the amino acid Thr794
Source: Cell Prolif. 2019 Nov 20;53(1):e12732. doi: 10.1111/cpr.12732 (PMC6985666; doi:10.1111/cpr.12732)
Supplement: Supplementary file 1 [file CPR-53-e12732-s001.docx]

**Supplementary Figure S1**


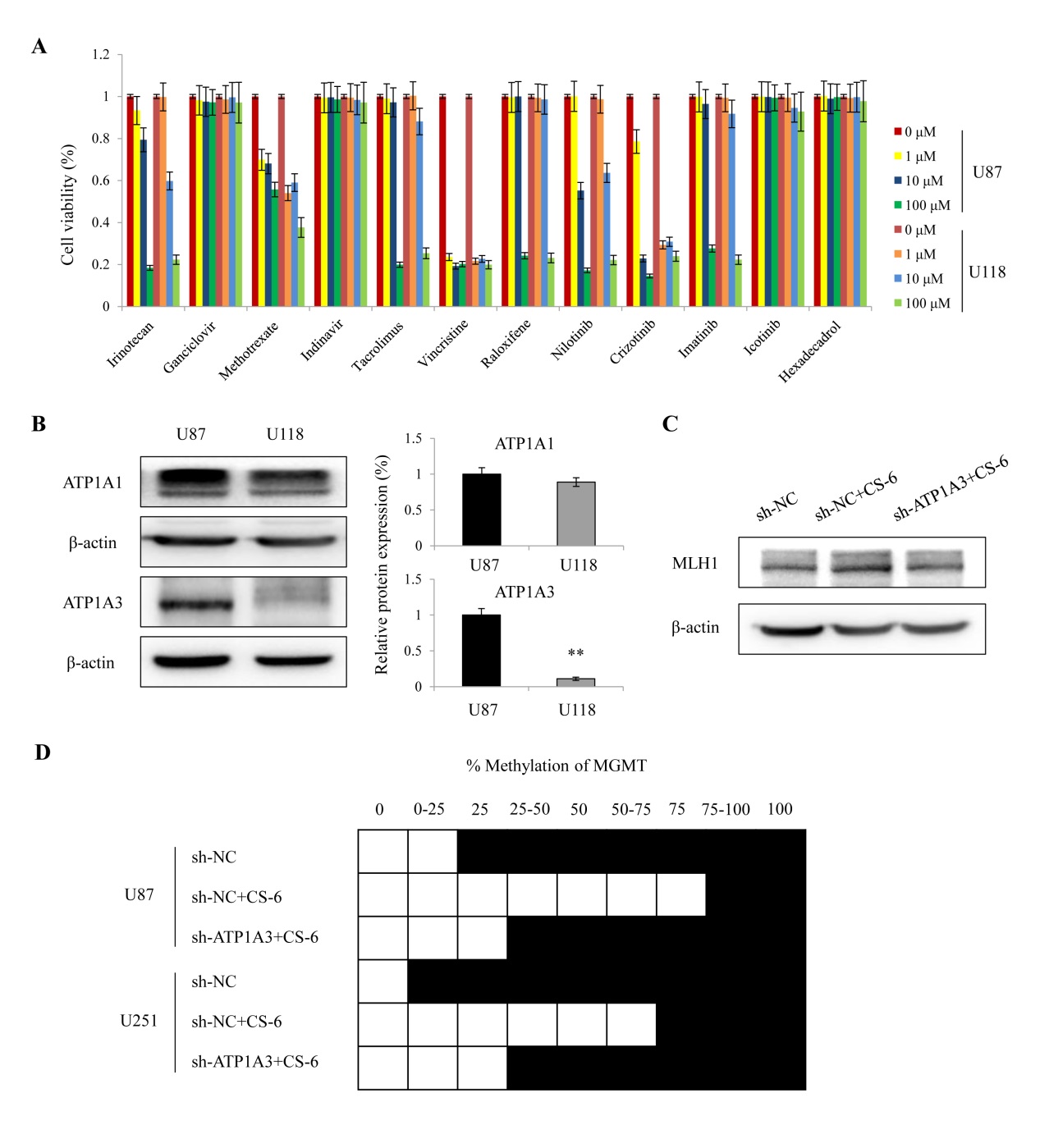


Figure S1. (A) Cell viability assay for testing the antiglioma efficacy of 12 commonly used clinical anticancer drugs. The concentrations 1 μΜ/L, 10 μΜ/L and 100 μΜ/L were used as indicated, and no significant difference in drug sensitivity between U87 and U118 cells was found. (B) The protein levels of ATP1A1 or ATP1A3 in U87 and U118 cells. (C) Expression of the mismatch repair (MMR) protein MLH1. MLH1 expression was significantly increased upon CS-6 treatment compared to that of the control group, while silencing ATP1A3 reversed this change. (D) The effect of CS-6 on MGMT methylation status, as well as the effect of ATP1A3 on CS-6-induced changes in MGMT methylation status in GBM.
