# Supplementary material for: Gamabufotalin induces a negative feedback loop connecting ATP1A3 expression and the AQP4 pathway to promote temozolomide sensitivity in glioblastoma cells by targeting the amino acid Thr794
Source: Cell Prolif. 2019 Nov 20;53(1):e12732. doi: 10.1111/cpr.12732 (PMC6985666; doi:10.1111/cpr.12732)
Supplement: Supplementary file 2 [file CPR-53-e12732-s002.docx]

**Supplementary Figure S2**


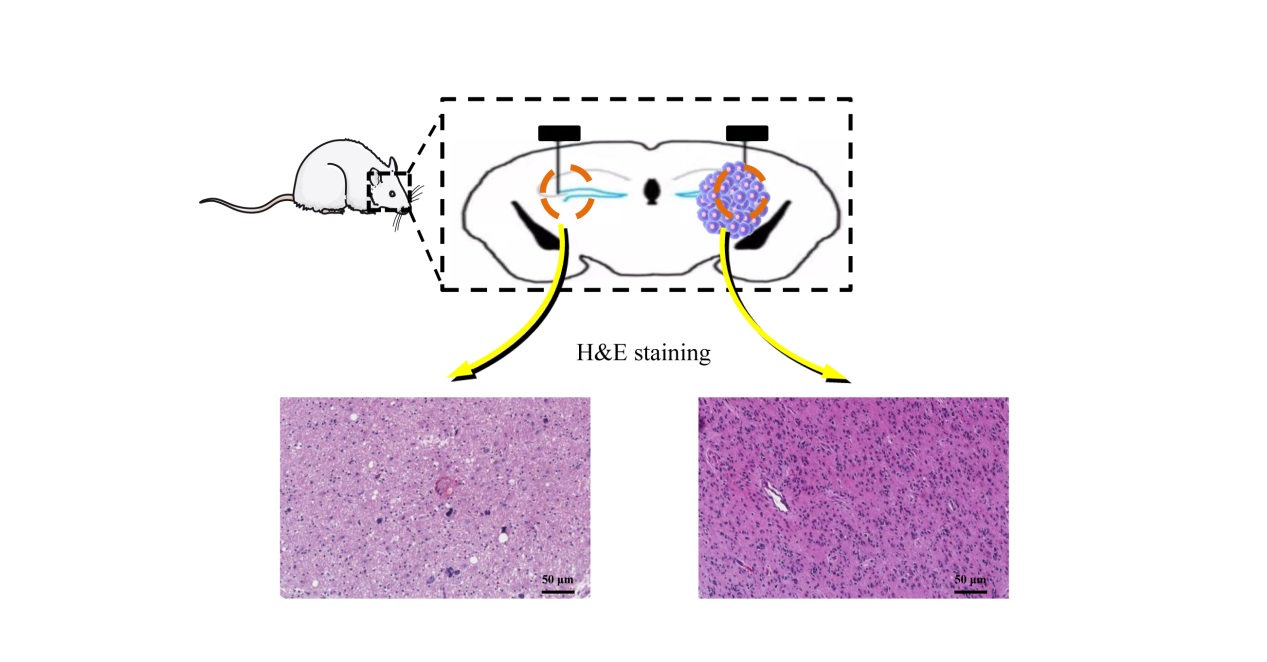


Figure S2. H&E staining was used for confirming normal brain tissues or orthotopic xenografts. In normal brain tissues, no obvious binuclear kernels and mitosis were observed, the nucleoli were smaller, and the morphology of the cells was more regular. However, the cells in tumor tissues were irregular in shape, had abundant cytoplasm and an abnormal nucleus, and had a high nucleus to cytoplasm ratio. There was obvious nuclear pleomorphism and nucleoli, as well as binuclear and mitotic phenomena. Scale bars, 50 μm.
