# Supplementary material for: Gamabufotalin induces a negative feedback loop connecting ATP1A3 expression and the AQP4 pathway to promote temozolomide sensitivity in glioblastoma cells by targeting the amino acid Thr794
Source: Cell Prolif. 2019 Nov 20;53(1):e12732. doi: 10.1111/cpr.12732 (PMC6985666; doi:10.1111/cpr.12732)
Supplement: Supplementary file 3 [file CPR-53-e12732-s003.docx]

**Supplementary Figure S3**

**
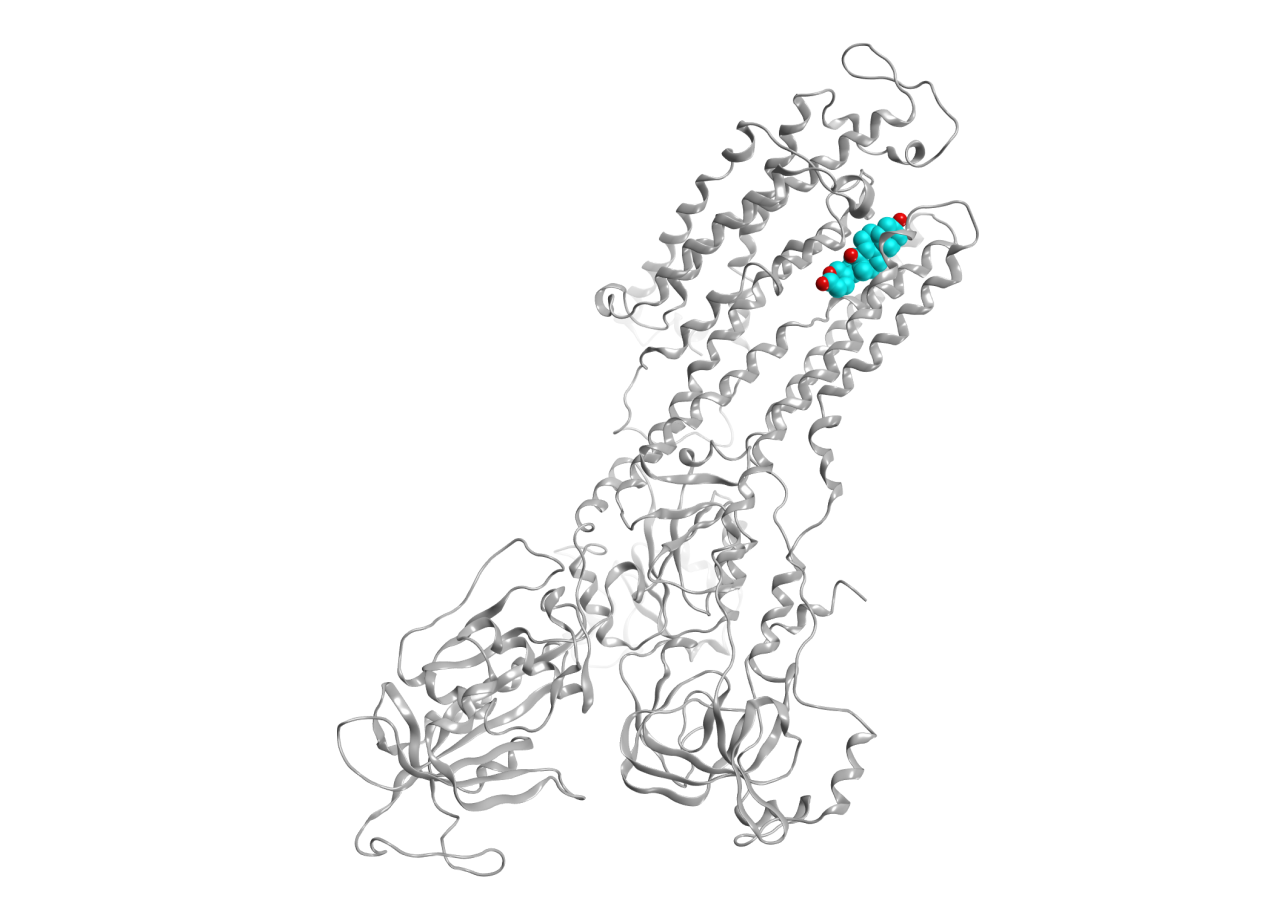
**

**
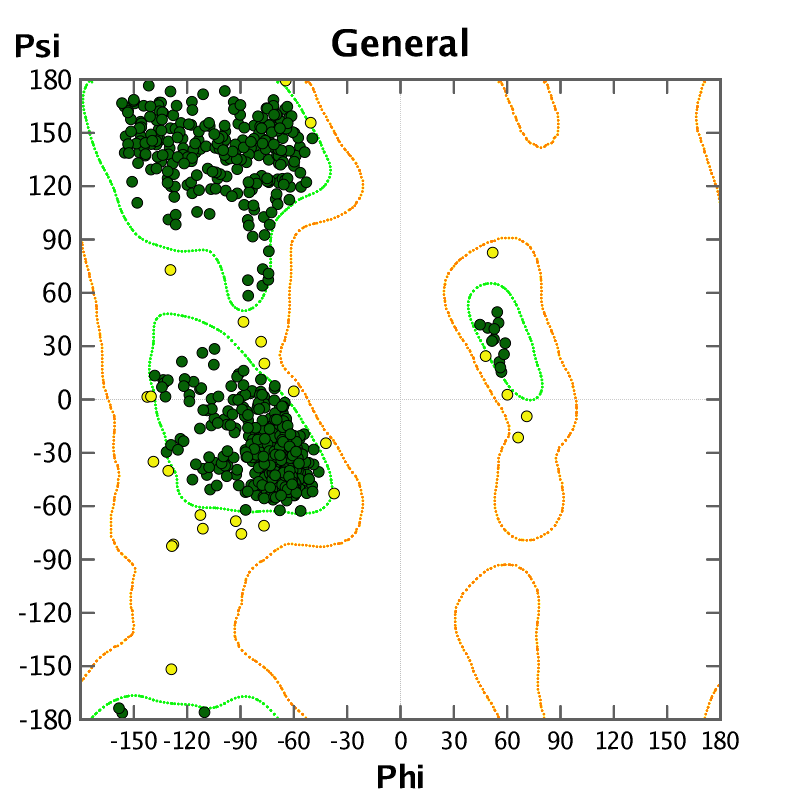
**

Figure S3. Top: backbone of the AT1A3 model. The AT1A3 backbone is shown in gray ribbons, and the ligand binding site is shown in the cyan space-filling model. Bottom: Ramachandran plot of the AT1A3 model. Dark green dots represent the residues in favored regions; yellow dots represent the residues in allowed regions; red dots represent the residues in irrational regions.
