# Supplementary material for: Gamabufotalin induces a negative feedback loop connecting ATP1A3 expression and the AQP4 pathway to promote temozolomide sensitivity in glioblastoma cells by targeting the amino acid Thr794
Source: Cell Prolif. 2019 Nov 20;53(1):e12732. doi: 10.1111/cpr.12732 (PMC6985666; doi:10.1111/cpr.12732)
Supplement: Supplementary file 4 [file CPR-53-e12732-s004.docx]

**Supplementary Figure S4**


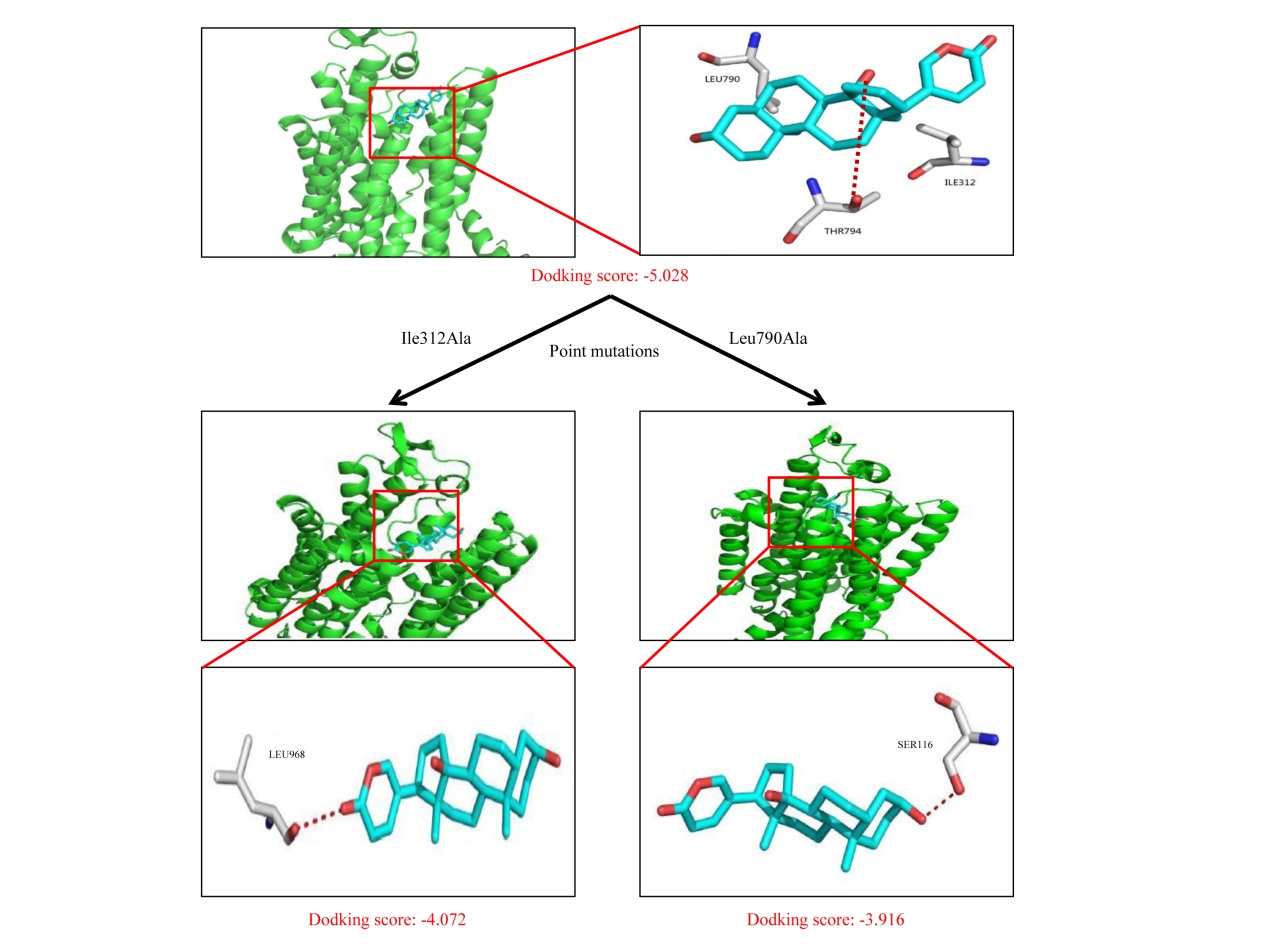


Figure S4. The details of the predicted binding mode of CS-6 and wild type ATP1A3 protein (upper panel) or ATP1A3 protein with the indicated point mutations (lower panel) using Schrödinger. The docking score for wild type ATP1A3 was -5.028, while the score for ATP1A3 with the Ile312Ala mutation was -4.072, and the score for ATP1A3 with the Leu790Ala mutation was -3.916, indicating that the binding strength between mutated ATP1A3 and CS-6 was significantly weaker than that between wild type ATP1A3 and CS-6.
