# Supplementary material for: Gamabufotalin induces a negative feedback loop connecting ATP1A3 expression and the AQP4 pathway to promote temozolomide sensitivity in glioblastoma cells by targeting the amino acid Thr794
Source: Cell Prolif. 2019 Nov 20;53(1):e12732. doi: 10.1111/cpr.12732 (PMC6985666; doi:10.1111/cpr.12732)
Supplement: Supplementary file 5 [file CPR-53-e12732-s005.docx]

**Supplementary Figure S5**


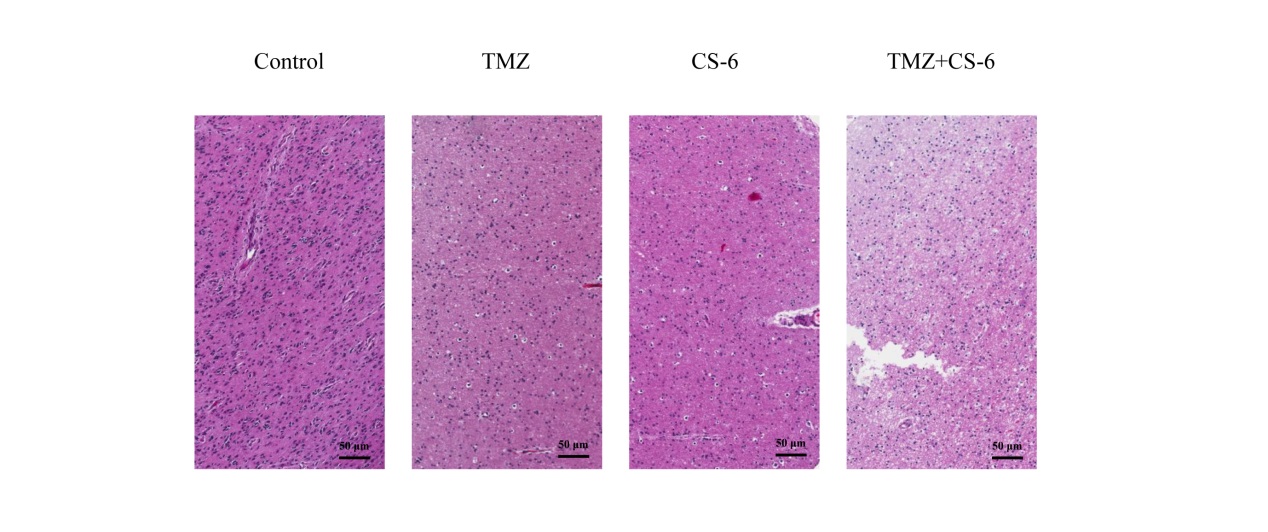


Figure S5. H&E staining demonstrating the features of the tumors in different groups. The results indicated that the tumor cells in the control group were irregular in shape, had abundant cytoplasm and an abnormal nucleus, and had a high nucleus to cytoplasm ratio. There was obvious nuclear pleomorphism and nucleoli. Binuclear and mitotic phenomena were also observed in the control group. However, after treatment with CS-6 or TMZ, especially the addition of CS-6 to TMZ, the nucleoli were smaller, and the morphology of the cells was more regular. Scale bars, 50 μm.
