# Supplementary material for: Gamabufotalin induces a negative feedback loop connecting ATP1A3 expression and the AQP4 pathway to promote temozolomide sensitivity in glioblastoma cells by targeting the amino acid Thr794
Source: Cell Prolif. 2019 Nov 20;53(1):e12732. doi: 10.1111/cpr.12732 (PMC6985666; doi:10.1111/cpr.12732)
Supplement: Supplementary file 6 [file CPR-53-e12732-s006.docx]

**Table S1.** Parameters involved in the experimental process.

| **Project** | **parameter** |
| --- | --- |
| **Chip** | 3D UVC chip |
| **Conditions of spotting** | 25℃, humidity：45.7% |
| **Temperature testing** | 20℃ |
| **Solution buffer** | 1×PBS (pH=7.4) |
| **Regeneration buffer** | Glycine·HCl (pH=2.0) |
| **Association reaction** | 300 s, 2 μL·s^-1^ |
| **Dissociation reaction** | 300 s, 2 μL·s^-1^ |
| **Enzymolysis reagent** | Trypsin (V5113) |
